# KiwiC for Vitality: Results of a Placebo-Controlled Trial Testing the Effects of Kiwifruit or Vitamin C Tablets on Vitality in Adults with Low Vitamin C Levels

**DOI:** 10.3390/nu12092898

**Published:** 2020-09-22

**Authors:** Tamlin S. Conner, Benjamin D. Fletcher, Jillian J. Haszard, Juliet M. Pullar, Emma Spencer, Louise A. Mainvil, Margreet C. M. Vissers

**Affiliations:** 1 Department of Psychology, University of Otago, Dunedin 9054, New Zealand; ben.fletcher@postgrad.otago.ac.nz; 2 Centre for Free Radical Research, Department of Pathology and Biomedical Science, University of Otago, Christchurch 8140, New Zealand; jill.haszard@otago.ac.nz (J.J.H.); juliet.pullar@otago.ac.nz (J.M.P.); margreet.vissers@otago.ac.nz (M.C.M.V.); 3 Department of Pathology and Biomedical Science, University of Otago, Christchurch 8140, New Zealand; emma.spencer@otago.ac.nz; 4 Department of Human Nutrition, University of Otago, Dunedin 9054, New Zealand; louise.mainvil@icloud.com

**Keywords:** Vitamin C status, energy, well-being, kiwifruit, mood

## Abstract

Consumption of vitamin C-rich fruits and vegetables has been associated with greater feelings of vitality. However, these associations have rarely been tested in experimental trials. The aim of the current study was to test the effects of eating a vitamin C-rich food (kiwifruit) on subjective vitality and whether effects are driven by vitamin C. Young adults (*n* = 167, 61.1% female, aged 18–35 years) with plasma vitamin C < 40 µmol/L were allocated to three intervention conditions: kiwifruit (2 SunGold™ kiwifruit/day), vitamin C (250 mg tablet/day), placebo (1 tablet/day). The trial consisted of a two-week lead-in, four-week intervention, and two-week washout. Plasma vitamin C and vitality questionnaires (total mood disturbance, fatigue, and well-being) were measured fortnightly. Self-reported sleep quality and physical activity were measured every second day through smartphone surveys. Nutritional confounds were assessed using a three-day food diary during each study phase. Plasma vitamin C reached saturation levels within two weeks for the kiwifruit and vitamin C groups. Participants consuming kiwifruit showed a trend of improvement in mood disturbance, significantly decreased fatigue, and significantly improved well-being after two weeks of the intervention. Improvements in well-being remained elevated through washout. Consumption of vitamin C tablets alone was associated with improved well-being after two weeks, and additionally improved mood and fatigue for participants with consistently low vitamin C levels during lead-in. Diet records showed that participants consuming kiwifruit reduced their fat intake during the intervention period. Intervention effects remained significant when adjusting for condition allocation groupings, age, and ethnicity, and were not explained by sleep quality, physical activity, BMI, or other dietary patterns, including fat intake. There were no changes in plasma vitamin C status or vitality in the placebo group. Whole-food consumption of kiwifruit was associated with improved subjective vitality in adults with low vitamin C status. Similar, but not identical changes were found for vitamin C tablets, suggesting that additional properties of kiwifruit may contribute to improved vitality.

## 1. Introduction

There is growing evidence that increased consumption of fruit and vegetables is associated with subjective feelings of vitality, which constitutes feelings of less fatigue, improved mood, and a “zest for life” [1,2,3], in conjunction with decreased levels of depression, anxiety, and stress [2,4,5]. Fruit and vegetables are rich in numerous essential nutrients and are the main source of vitamin C for humans and other species that cannot synthesize this compound in the liver [6,7]. Vitamin C availability determines intracellular levels throughout the body and saturation in most organs is achieved when plasma concentrations are >60 µmol/L [8,9,10]. Tissue vitamin C content is variable and some organs such as the brain and adrenals preferentially accumulate high concentrations of the vitamin even when plasma levels are below saturation [10,11]. High intracellular concentrations inside these organs coincide with a demand for ascorbate as a cofactor to support Cu- and Fe-containing enzymes, including those responsible for the synthesis of adrenalin, peptide hormones and collagen [12,13,14,15].

Insufficient intake of vitamin C results in lowered plasma ascorbate status. Levels below 11 µmol/L are indicative of the deficiency disease, scurvy [16,17], which is accompanied by impaired collagen synthesis resulting in skin changes, bruising, bleeding gums, and poor wound healing [7,18]. Early symptoms of vitamin C deficiency, including fatigue, depression, mental weariness, social introversion, hysteria, hypochondriasis, and reduced motivation and arousal [18,19,20,21,22], become apparent when plasma levels are <23 µmol/L, a condition described as hypovitaminosis C [20,21].

Several studies have suggested that increasing vitamin C intake to ensure plasma and tissue saturation can decrease the fatigue-related symptoms of vitamin C insufficiency and improve physical activity levels [16,23,24]. We, and others, have reported improvements in positive mood and vitality in association with increased fruit and vegetable intake [3,25,26,27,28]. Improved vitamin C status is closely aligned with increased fruit and vegetable consumption [29,30,31] and, given the role of vitamin C in neurotransmitter, norepinephrine, and peptide hormone synthesis [12,13,14,15], it is possible that many of the beneficial effects of fruit and vegetables could be attributed to this component. To determine the contribution of vitamin C to a food-related health benefit requires a specifically targeted and well-controlled study and this was the primary aim of our placebo-controlled intervention study.

We have previously reported pilot evidence of vitality improvements in young men with low baseline plasma vitamin C levels following a six-week intervention with kiwifruit, a high vitamin C food [32]. Although kiwifruit consumption resulted in the expected plasma saturation, we could not determine the relative contributions of the whole fruit or the vitamin C component to the observed decreased fatigue and improved vigour scores [32]. The current study was designed as a placebo-controlled trial to investigate the effects of increased vitamin C consumption via a food (kiwifruit), or tablets, on vitality changes in a sample of healthy young adults with low baseline vitamin C levels. The primary vitality outcome measures were self-reported mood disturbance, fatigue, and well-being, which were measured fortnightly during the study. Smartphone surveys were also administered every second night during the study to track day-level changes in sleep and physical activity while also minimising retrospective recall. We hypothesised that participants who consumed kiwifruit or vitamin C tablets would show greater improvements in their mood, fatigue, and well-being over time, relative to those who consumed placebo tablets. As blood plasma vitamin C levels were monitored fortnightly throughout the study, we also hypothesised that changes in vitamin C levels would mirror the improved psychological outcomes reported from consumption of kiwifruit or vitamin C tablets, relative to placebo.

## 2. Materials and Methods 

### 2.1. Trial Design

**Figure 1:**
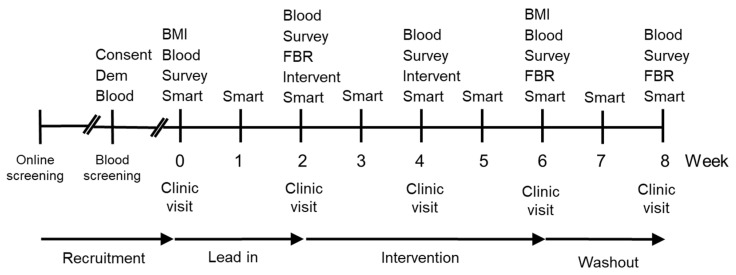
Timeline of study design. Key Consent-Consent form and study information; Dem-Demographic questionnaire; Blood-Blood sample; BMI-Body mass index; Survey–Survey measuring mood disturbance (POMS), fatigue (MFSI-SF), and well-being (WEMWBS); Smart-Smartphone survey, every second day during week; FBR–3-day Food and Beverage Record is returned; Intervent–Intervention provided with a two week supply of supplement (placebo, vitamin C, or kiwifruit).

The study was preregistered with the Australian and New Zealand Clinical Trial Registry (Trial ID: ACTRN12617001031358) and approved by the New Zealand Health and Disability Ethics Committee (17/NTB/104). The study was a three-armed, randomized (1:1:1 ratio), parallel arm, placebo-controlled trial (Figure 1). Participants were selected for the study following extensive screening that included measurement of plasma vitamin C status (see Screening section). The baseline plasma vitamin C status and vitality for enrolled participants was assessed before and after a two-week lead-in period. During a four-week intervention period the participants consumed, daily, either two SunGold™ kiwifruit, a chewable vitamin C tablet, or a chewable placebo tablet matched for appearance and flavour. A wash-out period established whether participants returned to baseline following the intervention. Plasma vitamin C levels and vitality outcomes were measured fortnightly across the study.

### 2.2. Participant Recruitment and Screening

**Figure 2:**
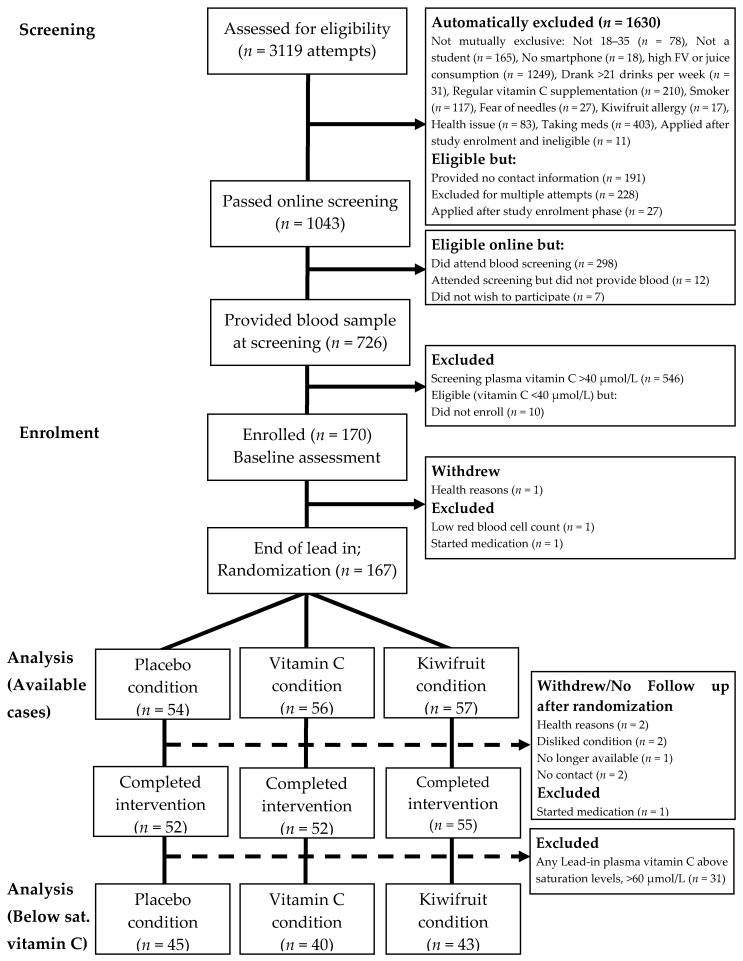
Participant flow diagram illustrating recruitment processes, inclusions and reasons for exclusion, allocated intervention condition, and withdrawals/loss-to-follow up. Below sat. vitamin C = Below saturation vitamin C status [selecting participants with vitamin C levels <60 µmol/L].

Participants were recruited and enrolled from university and polytechnic campuses in Dunedin, New Zealand, between July 2017 and April 2018. Eligibility criteria shown in Table 1 were used to recruit a cohort of young adults with low baseline vitamin C status. Participants completed an online screening questionnaire that accessed all eligibility criteria (except for vitamin C levels) prior to being invited to a nutrition clinic at the University of Otago where a registered nurse took blood samples to determine participants’ vitamin C levels. The flow of participants is represented in Figure 2. We screened 726 eligible individuals, measuring plasma vitamin C levels from fasting blood samples. Participants with plasma vitamin C concentration of ≤40 µmol/L were invited into the study (*n* = 180) and subsequently enrolled (*n* = 170) and randomized to a treatment arm (*n* = 167).

### 2.3. Condition Allocation

Our intention was to avoid participants being exposed to the other conditions during allocation of the intervention (e.g., participants seeing others receiving kiwifruit when they received tablets or vice versa). This was achieved by group randomisation based on scheduled clinic, either on separate days or across days, to ensure that everyone attending the same clinic would receive either kiwifruit or tablets. Participants receiving tablets at a given clinic were typically a random mix of the active and placebo groups. The allocation of clinics was determined by the lead author using a random number generator. As allocation occurred after participants were already enrolled into their clinic day (usually Mon–Thurs), the clinical study co-ordinator and prospective participants were unaware of their allocation when booking clinic appointments and did not learn of their allocation until they attended the clinic. A delay in the delivery of tablets meant that clinics in the first and second weeks were non-randomly allocated to the kiwifruit condition, and those in the third and fourth weeks, to the tablet conditions (randomised to placebo or vitamin C), with the remaining clinics randomised as intended. A randomisation schedule is shown in Supplementary Figure S1. Randomisation groups were accounted for in the statistical analyses (see Statistical Analyses, below). Tablets were bottled and labelled by the lead author (TSC) with the label including only the participant’s first and last name and tablet instructions to “chew one tablet daily, store in a dark dry place, and return bottle and unused tablets on next visit”. All research assistants and participants were double- blinded to their tablet condition. It was not possible to blind research assistants or participants to the kiwifruit condition. The separation of allocation clinics, however, meant that these participants were unaware of the nature of the other treatment conditions. Condition information was kept in an electronic password-protected document by the lead author (TSC) and unblinded following data collection and entry.

### 2.4. Procedure

Participants enrolled in the study attended one clinic session every two weeks during the eight-week study (5 clinic visits). At clinic visit 1 of the study, height, and weight were recorded to calculate BMI (kg/m^2^), a fasting blood sample was taken for vitamin C analysis, and the participants completed the vitality questionnaires. During clinic visits 2 and 3, participants were provided with a two-week supply of placebo tablets, vitamin C tablets, or kiwifruit. In addition, a comprehensive three-day food and beverage record was distributed during clinic visits 1, 3, and 4, and returned at the following clinic visits. Weight was also recorded following the intervention to calculate post-intervention BMI. See Figure 1 for the study timeline.

Throughout the entire eight-week trial, participants were sent brief smartphone surveys every alternate night to measure secondary covariates of self-reported sleep and physical activity, plus other outcomes not discussed in this report. A total of 29 smartphone surveys were sent to participants every other night around 6 p.m. for the duration of the study, starting the day of their first clinic visit and ending on the day of their last clinic visit. They were asked to respond to the survey that night before going to bed. If participants missed a smartphone survey, they were sent an email the following evening, which included a link to complete a make-up smartphone survey for the following day. If another consecutive survey was missed, a research assistant called the participant to confirm that there were no technical issues, and to ensure that the participant was still happy to participate.

### 2.5. Intervention

Participants were supplemented with either two SunGold™ kiwifruit per day, chewable vitamin C tablets, or chewable placebo tablets, for four weeks. SunGold™ kiwifruit contain 160 ± 31 mg of vitamin C per 100 g (Mean ± SD), and two SunGold™ kiwifruit (~150g combined weight) contain a total of approximately 250 mg of vitamin C [33]. To help preserve the SunGold™ kiwifruit, participants were instructed to store their kiwifruit in their refrigerator. Our independent analyses indicated that storage conditions did not affect the vitamin C content of the fruit. Each vitamin C tablet contained 250 mg of vitamin C and was identical in flavour and appearance with placebo tablets that contained no active vitamin C ingredients (both manufactured by Tishcon Corporation, Salisbury, MD, USA). Participants were instructed to store their tablets in a cool, dry place, such as in a cupboard.

### 2.6. Demographic and Health Characteristics

Demographic measures collected at the onset of the study included age, gender (male, female, and gender diverse), ethnicity (New Zealand Census categories, multiple endorsements allowed), year at university (1 to 6), and current socioeconomic status (measured by three items (“I have enough money to buy things I want”, “I don’t need to worry too much about paying my bills”, and “I don’t think I’ll have to worry about money too much in the future”), answered from 1 (strongly disagree) to 7 (strongly agree) (averaged, α = 0.79) [34]). Health information collected at the onset of the study included past and current smoking status, typical weekly alcohol use, typical daily fruit and vegetable consumption (“How many days a week do you eat fruit/vegetables?” (0 to 7 days), “On days you eat fruit/vegetables, how many servings do you usually eat?” (0.5 to 6+ servings), multiplied and divided by 7).

### 2.7. Primary Outcome Vitality Measures

The fortnightly survey consisted of three validated scales that measured experiences of vitality “during the past week, including today”. The Profile of Mood States questionnaire short form (POMS-SF) [35] is a 35-item measure where mood items are rated on a five-point Likert-type scale ranging from 0 (not at all) to 4 (extremely). The mood items reflect six factors of tension-anxiety, depression-dejection, anger-hostility, vigour-activity, fatigue-inertia, and confusion-bewilderment. Using these six factors, a total mood disturbance (TMD) was calculated (minimum to maximum score of −20 to 100) by summing the scores from the negative mood factors and subtracting the score from the vigor factor (Cronbach’s αs = 0.87–0.92). The Multidimensional Fatigue Symptom Inventory—short form (MFSI-SF) [36] is a 30-item measure that assesses general fatigue, physical fatigue, emotional fatigue, mental fatigue, and vigour (each with a maximum score of 24), rated on a five-point Likert-type scale ranging from 0 (not at all) to 4 (extremely). The vigour score was deducted from remaining sum scores to give a total fatigue score (minimum to maximum score of −24 to 96) (αs =0.89–0.96 across the 5 time points). The Warwick–Edinburgh Mental Well-being Scale (WEMWBS) [37] comprises 14 positive psychological well-being and vitality statements, which include feelings of cheerfulness, interest in new things, and spare energy levels, rated on a five-point Likert-type scale ranging from 1 (none of the time) to 5 (all of the time). All 14 items were summed to give a total well-being score (minimum to maximum score of 14 to 70) (αs = 0.91–0.93).

### 2.8. Smartphone Surveys

Automated smartphone surveys asked participants questions about their sleep and physical activity as secondary covariates. Sleep duration was measured using the single question “Approximately how many hours did you sleep last night?”, participants answered using a pulldown menu on their smartphone, which ranged from 0 to 24 h, increasing in increments of 30 min. Sleep quality was assessed with a single question on a 10-point Likert scale, “Please rate the quality of your sleep last night: 0 (“worst possible sleep”) to 10 (“best possible sleep”)”. Physical activity was assessed with the single item activity questionnaire, “Today, have you done a total of 30 min or more of physical activity, enough to raise your breathing rate? (sport, exercise, brisk walking, or cycling)”, answered with a “yes” (1) or a “no” (0).

### 2.9. Dietary Intake

Participants completed a diet and beverage record on three non-consecutive days, over a two-week period, at each phase of the study (lead-in, intervention, and washout). Diet records were used to calculate macronutrients to determine if dietary intake was equivalent between conditions over time. The food and beverage records were calculated using Diet Cruncher software (version 1.6, Way Down South Software, Dunedin, New Zealand) and the New Zealand FOODfiles Food Composition Database (2006).

### 2.10. Vitamin C Analysis

Fasting blood samples were kept on ice and processed within 2 h of collection as previously described [38]. Briefly, blood samples were centrifuged at 1000× *g* for 10 min at 4 °C, 700 μL of plasma removed and mixed with 700 µL cold 0.54 mol/L perchloric acid/DTPA solution, vortexed, and placed on ice for ten minutes. Precipitated protein was removed by centrifugation at 13,000× *g* for two minutes, and two samples of 500 μL supernatant stored at −80 °C. Samples were incubated with the reducing agent tris(2-carboxyethyl)phosphine hydrochloride (TCEP) to convert any oxidised ascorbate to the reduced form and vitamin C content was determined by high performance liquid chromatography (HPLC) with electrochemical detection [38,39]. Vitamin C concentration was calculated relative to an ascorbic acid standard curve run on the same day.

### 2.11. Missing Data Treatment and Statistical Analyses

Data were analysed with SPSS (version 26) and GraphPad Prism (version 8) with the alpha level set at 0.05 and two-tailed tests of significance. All participants randomized (*n* = 167) to a condition were included in the analyses following the “once randomized, always analysed principle” [40]. Specifically, participants from the randomized sample (*n* = 167) with any data at any point were included in the descriptive statistics, figures, and statistical analyses, reflecting an available cases approach, using all observed data with pairwise deletion only. We used this available cases approach with recommended sensitivity analysis comparisons because the amount of missing data was relatively small (3.36% missing data values) and below the 5% missingness rule-of-thumb [41,42,43]. Missing data were mainly due to drop out over the study duration, resulting in a monotone pattern, although this was broken by a general pattern of missingness for some data. Our data were determined to be not missing completely at random (Little’s MCAR test = χ^2^ (490) = 597.57, *p* = 0.001). Due to mental health outcomes being the primary measure, there was some concern that individuals with poorer mental health may be more likely to drop out of the study resulting in data missing not at random (MNAR). There was no evidence for this concern because the baseline distributions in total mood disturbance, fatigue, and well-being between participants who completed washout and participants who dropped out were relatively similar. Thus, we proceeded under the missing at random assumption (MAR) and completed sensitivity analysis to test the robustness of assumptions.

Descriptive statistics for participant characteristics were computed and the study outcome measures were plotted over time for each condition (vitamin C, total mood disturbance, fatigue, and well-being across weeks 0, 2, 4, 6, and 8 for the three conditions). Differences between outcome measures at baseline and intervention within each condition were determined with paired *t*-tests, comparing each intervention point at weeks 4 and 6, and the washout point at week 8, with the baseline measure at week 2 (second lead-in). Differences between conditions between baseline and intervention time points were investigated with ANOVA (mixed ANOVAs with condition (placebo, vitamin C, and kiwifruit) and time (Study Week 2, 4; Study Week 2, 6; or, Study Week 2, 4, 6) as factors) and ANCOVA (adding to the above analysis of variance models any covariates reflecting significant between-condition differences in demographic, health, or dietary factors). One-way ANOVA was used to determine any demographic, health, or dietary differences between conditions at each phase of the study. Post hoc analyses with Tukey’s HSD (α = 0.05) were used to determine differences between conditions. If the homogeneity of variance was violated, then Welch’s F test and post hoc analyses with Games-Howell were conducted. All ANOVA analyses were repeated for sensitivity analysis comparisons to assess the robustness of the sample due to missing data. Using pattern mixture models, different distributions between participants who dropped out and who remained in the study were analysed (see Online Supplementary Material).

## 3. Results

As shown in Figure 2, a total of 170 participants enrolled in the study and attended the baseline session (Week 0). Three people withdrew or were excluded from the study prior to randomization, leaving 167 participants randomized into conditions and included in the primary available cases analysis. After randomization, five people never received their intervention (two for no contact/no show, both assigned to placebo condition; one withdrew/no longer available, assigned to vitamin C condition; two disliked assigned condition and were unwilling to adhere, one assigned to vitamin C condition and one assigned to kiwifruit condition). A further three people withdrew or were excluded for health or medical reasons prior to completing the four-week intervention period (two for health reasons, both in vitamin C condition; one started medication, in kiwifruit condition). Thus, a total of 159 participants completed the four-week intervention and 155 completed the full washout.

For more detail, see Table 2. These numbers reflect a post-randomization attrition rate of 4.79% for completing the four-week intervention (placebo = 3.70%; vitamin C = 7.14%; and kiwifruit = 3.51%), and 7.19% for completing the full study including washout (placebo = 5.56%; vitamin C = 8.93%; kiwifruit = 7.02%). Attrition rates did not differ by condition (*p*s = 0.599 and 0.760).

**Figure 3:**
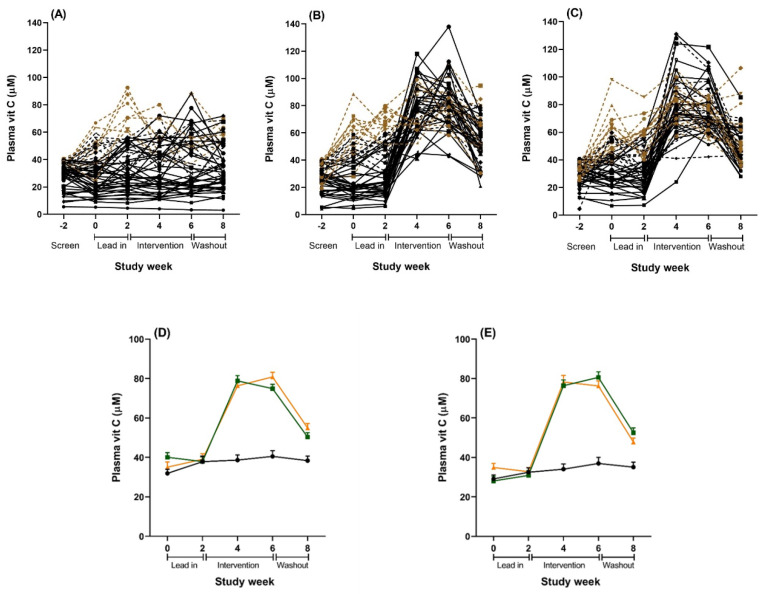
Plasma vitamin C concentrations over the study period for the total sample (*n* = 167). (**A**–**C**): Individual results for all participants randomized to (**A**) placebo, (**B**) vitamin C tablet, and (**C**) kiwifruit conditions. Solid black lines are individuals in the per protocol (PP) analyses with lead-in plasma vitamin C levels <40 µmol/L (*n* = 92). Combined black lines (solid and dotted) are individuals in the below saturation analyses with lead-in plasma vitamin C levels <60 µmol/L (*n* = 128). Brown dotted lines are individuals with lead-in plasma vitamin C levels >60 µmol/L excluded from the below saturation analysis (*n* = 39). (**D**): Combined results of all plasma vitamin C levels for the total sample (*n* = 167). Black = placebo tablet, *n* = 54; orange = vitamin C tablet, *n* = 56; green = kiwifruit, *n* = 57. (**E**): Combined results of all plasma samples for the cohort sub-set with vitamin C concentrations <60 µmol/L at baseline and lead in (*n* = 128). Black = placebo tablet, *n* = 45; orange = vitamin C tablet, *n* = 40; green = kiwifruit, *n* = 43. Data are the raw unadjusted means ± SE.

Repeated measurement of plasma vitamin C status during the lead-in period showed considerable variation in a number of individuals, with many presenting above initial recruitment levels (40 µmol/L) and some increasing to well above saturation levels (60 µmol/L) prior to intervention (Figure 3). This meant that some participants with adequate vitamin C levels were receiving the intervention. Therefore, we ran two additional subgroups analyses to try to address our original research hypothesis of testing the intervention on people with low vitamin C. We ran a per protocol (PP) analysis on 92 participants with plasma vitamin C level <40 µmol/L during the lead-in period, and a second analysis on 128 participants who maintained below saturation levels of vitamin C (<60 µmol/L) during the lead-in period, as this latter analysis achieved sufficient power and still assessed individuals with low vitamin C levels (a priori power analyses indicated that 120 participants (40 per condition) provided 80% power to detect an effect size of 0.7). Demographic characteristics for the PP subgroup (*n* = 92) and the below saturation vitamin C subgroup (*n* = 128) are reported in the Online Supplementary Material. For all analysis, participants were analysed according to their originally assigned condition.

The participant baseline characteristics for the sample overall and by condition are detailed in Table 3, with additional descriptive statistics in Supplemental Tables S1 and S2. The recruited cohort was 40/60% men/women, was ethnically heterogeneous and of slightly above average socio-economic status. The average BMI was 24 ± 4.4 (Mean ± SD), with most participants falling within the healthy range; range was from 13 to 43. The sample was not especially distressed as shown by the mean scores on the vitality measures from the two lead-in clinic visits (Supplemental Table S1). The average total mood disturbance reported at lead-in was relatively low (mean (*M*) = 5.68–5.38, SD = 14.12–16.63), with higher scores indicating worse mood on a scale from −20 to 100. Average fatigue was also relatively low (*M* = 2.45–1.18, SD = 14.24–15.57), with higher scores indicating higher fatigue on a scale from −24 to 96. Average well-being was moderate to high (*M* = 47.85–48.52, SD = 8.65–8.96), with higher scores indicating greater well-being on a scale from 14 to 70. The three conditions were mostly equivalent in demographic characteristics, baseline health characteristics, baseline vitality measures, and baseline vitamin C levels (ANOVAs or Chi-Squares not significant), except that the kiwifruit condition had younger participants (F(2.164) = 8.906, *p* < 0.001) with fewer years at university (F(2.164) = 3.305, *p* = 0.039) and a trend towards fewer Asian participants (χ^2^ (2.167) = 5.230, *p* = 0.073) than the other conditions. Moreover, the placebo condition had lower lead-in sleep quality than the vitamin C condition (F(2.163) = 3.054, *p* = 0.050). Accordingly, these variables were controlled for in ANCOVA.

Plasma vitamin C levels were significantly affected by both the kiwifruit and vitamin C tablet interventions and reached saturation levels (>60 µmol/L) within two weeks (Table 4 and Figure 3). The vitamin C levels were mostly equivalent for the kiwifruit and vitamin C conditions during weeks 2 to 8, except that the vitamin C condition had a trend towards higher levels of vitamin C than the kiwifruit condition at week 6 (81 vs. 75 µmol/L, t(105) = 1.860, *p* = 0.066). Levels were maintained at saturation for the duration of the intervention in both conditions. In contrast, there was no change in vitamin C status throughout the study for those in the placebo condition. Vitamin C levels for the intervention conditions decreased significantly following a two-week washout (Week 8) but were still higher than the baseline levels and the placebo group (both different from Week 2 at *p* < 0.001) (Figure 3).

**Figure 4:**
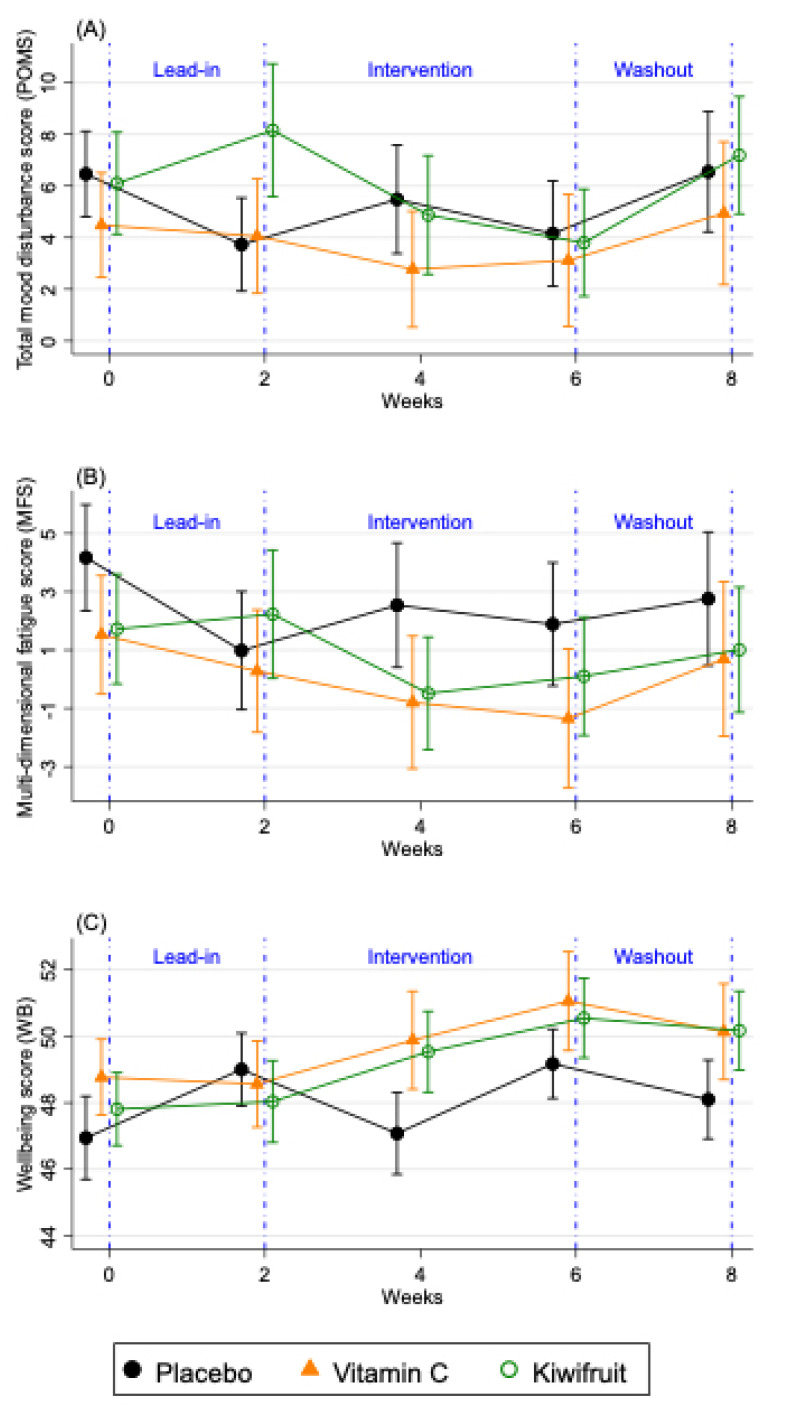
Changes in fortnightly (**A**) total mood disturbance scores (POMS), (**B**) multidimensional fatigue score (MFS), and (**C**) well-being (WB) over the study period for the total sample (*n* = 167). Results are presented as means ± SE for participants allocated to placebo tablet (black lines), vitamin C tablet (orange lines) and kiwifruit (green lines) conditions. Lead-in Week 2 served as baseline, which was compared against Week 4 and Week 6 of the intervention.

The changes in the primary vitality outcomes by condition for the total sample (*n* = 167) are shown in Figure 4 and Supplemental Table S1, which compares two weeks of intervention (Study Week 4), the end of the intervention (Study Week 6), and washout (Study Week 8), to the end of the lead-in period (Study Week 2). For the POMS total mood disturbance, only participants in the kiwifruit condition (Figure 4) showed significantly reduced total mood disturbance after two weeks of intervention (Study Week 4), and at the end of the intervention (Study Week 6) which returned to baseline at washout (Study Week 8). Participants in the vitamin C condition showed non-significant reductions in their POMS total mood disturbance (Figure 4) and those in the placebo condition were unchanged over time (Figure 4). Participants in the kiwifruit condition showed a trend reduction in fatigue after two weeks of supplementation (Figure 4) but not at four weeks of supplementation (Study Week 6). For participants in the vitamin C condition, fatigue increased significantly upon withdrawal of vitamin C during washout (*p* < 0.05). No changes in fatigue were seen in the placebo condition. For well-being, there was a significant increase in well-being after two weeks of kiwifruit supplementation, which remained elevated after four weeks of supplementation and did not decrease at washout (Figure 4). Participants in the vitamin C group also showed a trend increase in well-being at the end of the intervention (Figure 4), which was eliminated at washout. There was no change in well-being in the placebo group except for an increase between Study Week 4 and 6 of placebo supplementation, which was mainly due to well-being returning to baseline levels (Supplemental Table S1). There was no reported or observed harm or adverse events as a result of any intervention arm in this study.

The analysis of variance results is presented in Table 5. The available cases analysis (far right columns) showed significant condition x time effects when comparing Study Week 2 (end of lead-in) with Study Week 4 (second week of intervention) for POMS total mood disturbance (*p* = 0.035, partial eta squared (η_p_^2^) = 0.041) and well-being (*p* = 0.009, η_p_^2^ = 0.058) and a trend for fatigue (*p* = 0.063, η_p_^2^ = 0.034). These effects remained significant when controlling for the demographic and health covariates. However, there were no significant condition x time effects when comparing Study Week 2 (end of lead-in) with Study Week 6 (end of intervention; all ns), and there was only a trend for well-being when comparing all three time points together (*p* = 0.054, η_p_^2^ = 0.029). Results were similar in the adjusted models.

The intervention effects were not due to differences between conditions in BMI (Welch’s F(2.99.036) = 0.104, *p* = 0.901), physical activity (intervention weeks 3–4, F(2.158) = 0.285, *p* = 0.752; intervention weeks 5–6, F(2.157) = 0.029, *p* = 0.972), sleep quality (intervention weeks 3–4, F(2.158) = 0.657, *p* = 0.520; intervention weeks 5–6, F(2.157) = 0.643, *p* = 0.527), sleep quantity (intervention weeks 3–4, F(2.158) = 0.370, *p* = 0.692), dietary factors such as total protein intake (F (2.156) = 0.556, *p* = 0.575), or total carbohydrate intake (F (2.156) = 2.198, *p* = 0.114). There was a trend difference between conditions for sleep quantity (intervention weeks 5–6, F(2.157) = 2.496, *p* = 0.086), which was driven by a trend for participants in the vitamin C condition to sleep for marginally longer compared to the placebo condition (*p* = 0.095), but not the kiwifruit condition (*p* = 0.194). There was also a trend difference between conditions for total energy intake (KJ) (F (2.156) = 2.706, *p* = 0.070). A post hoc Tukey HSD test indicated a trend for lower total energy intake (KJ) for the kiwifruit condition during the second half of the intervention (weeks 5–6) compared to the vitamin C condition (*p* = 0.073). This difference between conditions was driven by total fat intake (Welch’s F (2.96.204) = 4.016, *p* = 0.021). A post hoc Games-Howell test indicated significantly lower total fat intake (grams) for participants in the kiwifruit condition during the second half of the intervention (weeks 5–6) compared to participants in the vitamin C condition (*p* = 0.019; Supplemental Table S2). Importantly, as shown in Table 5, adjusting for the difference in sleep quantity and fat intake did not change the condition × time effects.

Sensitivity analysis using pattern mixture models based on monotone missing data for the primary outcomes suggested that the observed cases analysis was mostly robust (see Online Supplementary Material). When looking at different patterns of missing data, the results maintained the same patterns (Supplemental Table S12). Sensitivity analysis of total mood disturbance, fatigue, and well-being did not weaken results.

The changes in the vitality outcomes by condition for the below saturation vitamin C sample (*n* = 128) are shown in Supplemental Table S5, Supplemental Figure S1, and Table 5. When testing individuals with consistently below saturation levels of vitamin C during the lead-in phase, participants in the vitamin C arm showed reductions in fatigue after supplementing with vitamin C for two weeks (*p* = 0.065) and four weeks (*p* = 0.017), which returned to baseline levels at washout (*p* = 0.379). ANOVA results from Table 5 also showed significant condition x time effects for fatigue in the low vitamin C group (*n* = 128). These patterns for fatigue were not observed when analysing the full sample suggesting that vitamin C benefits fatigue in those with low vitamin C levels. By contrast, the effects of kiwifruit were similar in the PP, below saturation vitamin C, and observed cases analyses and tended to occur early in the intervention at two weeks, whereas the effects from vitamin C alone tended to build up through the intervention. Results for the PP sample (*n* = 92) mostly mirrored results for the below saturation vitamin C sample and are shown in Supplemental Table S9, Supplemental Figure S2, Table 5 and Table 6.

## 4. Discussion

Our randomised controlled trial aimed to investigate the extent to which vitamin C contributes to the mood improvements reported in association with increased fruit and vegetable intake [3,25,26,27,28]. We were able to compare the effects of increased vitamin C intake from a fruit source (SunGold™ kiwifruit) with the equivalent amount of vitamin C in tablet form and with a placebo tablet on subjective vitality in healthy young adults. Improvements in mood and well-being were apparent for the group consuming kiwifruit, whereas improvements in fatigue and well-being were apparent for the group consuming vitamin C but only if they had low levels of vitamin C as selected in the below saturation vitamin C or per protocol subgroups analyses. Importantly, there were no changes in any outcomes in the placebo group.

These results indicate that the vitamin C content of the fruit may be a significant component contributing to improvements in vitality. However, the kiwifruit-mediated effects were broader (affecting more outcomes for more people), and occurred earlier in the intervention at two weeks, whereas the effects from vitamin C alone were narrower (affecting fewer outcomes for specific types of people) and tended to occur throughout the intervention. These differences are notable, particularly given that the kiwifruit and tablet interventions resulted in a comparable increase in plasma vitamin C levels and there are no known differences in uptake and bioavailability of “synthetic” versus “natural” vitamin C [44]. In this regard, our study provides novel insight into the potential differences between single-nutrient supplementation with a vitamin C tablet versus provision of the same content of vitamin C from a whole fruit. It appears that additional and maybe extended benefits result from whole fruit consumption. This finding reinforces the potential health and well-being benefit from whole food rather than single-nutrient dietary supplementation, although neither intervention showed any harmful side-effects.

Other compounds in whole kiwifruit that could account for their broader effects include fibre, folate, or potassium, all of which are relatively high in SunGold™ kiwifruit [33,45,46]. Increased dietary fibre intake can benefit the gut microbiome, which has been linked to changes in mental health [47,48,49]. Low folate intake has been associated with negative mental health outcomes, such as depression [50], and a low sodium and high potassium diet is associated with greater vigour and lower levels of depression, tension, and POMS total mood disturbance [51]. However, the relationship between dietary factors and mental health is complex and is likely mediated by several micronutrient interactions. Further research is required to determine the full impact that dietary factors have on mental health in isolation. Interestingly, we found that supplementing with kiwifruit was associated with reductions in the intake of dietary fat. This may be due to participants substituting kiwifruit for high-fat snacks and/or reductions in appetite from consuming a nutrient-dense whole food. Controlling for fat intake did not attenuate or eliminate the intervention effect, suggesting that reduced fat intake is unlikely to be driving the differences in vitality between the kiwifruit and vitamin C or placebo tablet groups.

Our study design had numerous strengths. The use of placebo and control groups is frequently problematic for food intervention studies and determination of the active ingredient in any particular food is challenging [52]. By assigning control arms that included a matched vitamin C tablet and a placebo tablet, we were able to include a blinded intervention (tablets) that avoided the complexities associated with the assignment of a different control food that can introduce potentially confounding nutrients. Use of the vitamin C tablet and placebo arms therefore assessed potential benefits of vitamin C on mental health in isolation to other dietary components [53]. Additional strengths included the length of the eight-week trial with lead-in and washout periods, extensive screening to identify people with low vitamin C levels that ensured that the intervention was appropriately targeted towards individuals in whom vitamin C supplementation would improve baseline status, strict inclusion and exclusion criteria to omit people with conditions that might affect vitamin C processing, and objective measurement of blood vitamin C levels at five time points.

At below-saturation levels, plasma vitamin C concentrations are very sensitive to changes in dietary intake [16]. Our observation that a number of individuals with an initial plasma concentration <40 µmol/L subsequently showed saturation levels >60 µmol/L during lead-in is consistent with a variable dietary intake for occasional fruit and vegetable eaters. This is potentially a limitation of the study. Running a per protocol (PP) and below saturation vitamin C analysis of those individuals whose plasma vitamin C levels were consistently low or below saturation during the lead-in period ensured that we could still understand how our intervention affected this susceptible population. This enabled us to show that single-nutrient supplementation with a vitamin C tablet was mainly beneficial in those below saturation levels of vitamin C. Further analysis of those with below saturation levels of vitamin C during lead-in allowed us to also assess the intervention on an adequately powered group (*n* = 128) in whom increases in vitamin C status was possible. The consistent monitoring of vitamin C levels is therefore a strength of this study and has enabled us to avoid the pitfalls of many other vitamin supplementation studies that have not monitored vitamin status, which potentially confounds interpretation [21,54]. Lastly, we noted a trend ethnicity imbalance between the kiwifruit group and the other conditions. Controlling for this imbalance reduced but did not eliminate our intervention effects, which suggest potential ethnicity-linked differences in the effects of vitamin C on vitality worthy of follow up. However, this remains an exploratory question for future research adequately powered to test for differences.

The similarities between the effects of vitamin C and kiwifruit on vitality outcomes suggests that the vitamin contributes to the beneficial effects of fruit consumption. This has often been suggested, but not directly tested. Vitamin C supports numerous functions in vivo that could account for these effects. It is an essential cofactor for two enzymes that synthesise carnitine that is necessary for fatty acid metabolism and energy production [55,56]. Vitamin C availability affects carnitine production in animals and humans and has been shown to affect energy production via fat metabolism in healthy individuals [24,55,57,58].

Vitamin C is also essential for the synthesis of neurotransmitters and peptide hormones in the nervous system and adrenal glands. The Cu-containing enzyme dopamine β-hydroxylase catalyses the conversion of dopamine to norepinephrine [14,59,60] and vitamin C deficiency results in decreased norepinephrine production in animals [61,62,63]. In addition, vitamin C is required for the regeneration of tetrahydrobiopterin, the enzyme cofactor for the tyrosine hydroxylase that produces dopamine [64]. Dopamine is a neurotransmitter important for contributing “zest to life” [65], suggesting a possible linking between vitamin C and vitality. The biosynthesis of many amidated peptide-based hormones, including oxytocin, vasopressin, thyrotropin-releasing hormone, and substance P, is catalysed by the vitamin C-dependent peptidylglycine α-amidating monooxygenase (PAM) [13,66,67,68,69]. Many of these hormones affect neural processes and could influence both physical and emotional well-being. In particular, oxytocin release is associated with many positive mood enhancing effects [70]. Strikingly, tissues where these catecholamines and peptide hormones are synthesised contain the highest levels of vitamin C in the body, i.e., pituitary, neurons, ovaries, testes, eyes, adrenals, placenta, thymus, and pancreas [13,69,71]. Based on the above research, it is clear that vitamin C has the potential to influence the nervous system.

However, it is also clear from our study that there are differences in effects between vitamin C alone and whole fruit. This is in line with other studies that have shown vitality gains with fruit and vegetable consumption independently of vitamin C status [25]. Together with the results from the current study, it would appear that there are indeed additional health benefits that are measurable in a healthy population and that can be gained from ensuring that essential micronutrients such as vitamin C are delivered through whole food rather than a supplement. Future research should also consider the wider mechanisms linking consumption of whole fruits and vegetables to subjective vitality.

## Figures and Tables

**Table 1:** Inclusion and exclusion criteria.

Inclusion Criteria (All Required for Inclusion)	Exclusion Criteria (Only One Required for Exclusion)
Males and females aged 18–35 years	Taking prescription medication (within past three months)
Plasma vitamin C levels <40 µmol/L	Allergy/intolerance to kiwifruit
Non-smoker	Recent smoker (within previous year)
Currently a student	Taking vitamin C supplements (within past three months)
	High fruit/juice & vegetable consumption (≥5 servings/day)
	Excessive alcohol consumption (>21 standard drinks/week)
	Diabetes mellitusBleeding disorders
	Fainting due to fear of needles

**Table 2:** Number of participants who completed the vitality outcome measures each visit.

		Condition		
	Total Sample	Placebo	Vitamin C	Kiwifruit
Week 0 (Lead in)	*n* = 170			
Randomization	*n* = 167	*n* = 54	*n* = 56	*n* = 57
Week 2 (Lead in)	*n* = 164	*n* = 52	*n* = 55	*n* = 57
Week 4 (Intervention)	*n* = 161	*n* = 52	*n* = 53	*n* = 56
Week 6 (Intervention)	*n* = 159	*n* = 52	*n* = 52	*n* = 55
Week 8 (Washout)	*n* = 155	*n* = 51	*n* = 51	*n* = 53

**Table 3:** Baseline characteristics for the total sample (*n* = 167) and each condition.

	Total Sample (*n* = 167)	Placebo (*n* = 54)	Vitamin C (*n* = 56)	Kiwifruit (*n* = 57)
Age (years)	21.69 (3.54)	21.87 (3.31)	22.95 (4.46)	20.28 (1.87)
Gender:				
Male	64 (38.3%)	20 (37.0%)	22 (39.3%)	22 (38.6%)
Female	102 (61.1%)	34 (63.0%)	34 (60.7%)	34 (59.6%)
Gender diverse	1 (0.6%)	0 (0.0%)	0 (0.0%)	1 (1.8%)
Ethnicity:				
European	64 (38.3%)	19 (35.2%)	19 (33.9%)	26 (45.6%)
Asian	67 (40.1%)	25 (46.3%)	26 (46.4%)	16 (28.1%)
Indian	15 (9.0%)	5 (9.3%)	6 (10.7%)	4 (7.0%)
Māori & Pasifika	9 (5.4%)	2 (3.7%)	2 (3.6%)	5 (8.8%)
Other & multiple	12 (7.2%)	3 (5.6%)	3 (5.4%)	6 (10.5%)
Year of study at university	2.80 (1.51)	2.81 (1.53)	3.16 (1.74)	2.44 (1.17)
Socioeconomic status (1 to 7) ^1^	4.64 (1.34)	4.81 (1.25)	4.58 (1.34)	4.52 (1.41)
Height (cm)	168.49 (10.31)	168.21 (11.09)	168.51 (9.91)	168.73 (10.10)
Weight (kg):				
Pre-intervention	68.01 (15.71)	67.72 (18.83)	67.82 (14.92)	68.47 (13.29)
Post-intervention ^2^	68.08 (15.89)	67.70 (19.12)	68.01 (15.12)	68.50 (13.30)
BMI (kg/m^2^):				
Pre-intervention	23.84 (4.44)	23.85 (5.73)	23.70 (3.76)	23.98 (3.65)
Post-intervention ^2^	23.88 (4.44)	24.00 (5.86)	23.66 (3.66)	23.95 (3.53)
No History of Smoking:	150 (89.8%)	49 (90.7%)	49 (87.5%)	52 (91.2%)
Vegetables servings/day	1.00 (0.756)	0.97 (0.80)	0.99 (0.72)	1.02 (0.76)
Fruit servings/day	0.39 (0.38)	0.37 (0.36)	0.38 (0.32)	0.43 (0.46)
Alcoholic standards /week	3.43 (5.23)	2.60 (4.02)	3.04 (5.97)	4.59 (5.36)
Sleep duration (hours) ^3^	7.18 (1.04)	7.07 (1.11)	7.35 (0.86)	7.11 (1.11)
Sleep quality (1 to 10) ^3^	6.54 (1.29)	6.21 (1.42)	6.80 (1.29)	6.61 (1.12)
Physical activity (30 min/ day):				
% of days ^3^	46.1%	47.5%	45.5%	45.5%

^1^ *n* = 165; Placebo *n* = 54; Vitamin C *n* = 56; Kiwifruit *n* = 55 ^2^ *n* = 158; Placebo *n* = 52; Vitamin C *n* = 51; Kiwifruit *n* = 55. ^3^ *n* = 166; Placebo *n* = 54; Vitamin C *n* = 55; Kiwifruit *n* = 57.

**Table 4:** Total sample plasma vitamin C concentrations (µmol/L) by condition during the study period.

	Placebo		Vitamin C		Kiwifruit	
	*n*	Mean (SD)	*n*	Mean (SD)	*n*	Mean (SD)
Week 0 (Lead-in)	54	31.93 (15.33)	56	35.10 (19.6)	56	40.08 (17.7)
Week 2 (Lead-in)	52	38.19 (20.82)	55	39.10 (20.96)	57	37.90 (17.88)
Week 4 (Intervention)	52	36.82 (18.89)	53	76.34 (17.57) ****^a^	55	78.91 (20.21) ****^a^
Week 6 (Intervention)	52	39.25 (20.82)	52	80.84 (17.14) ****^a^	55	74.88 (16.03) ****^a^ *^b^
Week 8 (Washout)	51	38.27 (17.66)	51	54.94 (16.52) ****^abc^	53	50.35 (16.15) ****^abc^

Note. Results shown are means (standard deviations). ^a^ Comparison with Week 2 (Lead-in); ^b^ Comparison with Week 4 (Intervention); ^c^ Comparison with Week 6 (Intervention). * *p* < 0.05, **** *p* < 0.0001.

**Table 5:** Effect of Vitamin C tablet or Kiwifruit on mood disturbance, fatigue, and well-being after 2 and 4 weeks of intervention, compared to week 2 of baseline (end of Lead in) (*n* = 161).

	Placebo Group (*n* = 52)		Vitamin C Tablet Group (*n* = 53)				Kiwifruit Group (*n* = 56)			
	Baseline Mean (SD)	Mean Change from Baseline (SD)	Baseline Mean (SD)	Mean Change from Baseline (SD)	Mean Difference in Change (95% CI) Compared to Placebo ^a^	*p*-Value	Baseline Mean (SD)	Mean Change from Baseline (SD)	Mean Difference in Change (95% CI) Compared to Placebo ^a^	*p*- Value
POMS total score ^b^										
2 weeks of intervention	3.7 (13.0)	1.8 (13.1)	3.9 (16.7)	−1.1 (10.1)	−3.0 (−6.9, 0.8)	0.121	8.6 (19.4)	−3.7 (8.9)	−4.0 (−8.1, 0.2)	0.062
4 weeks of intervention ^c^	3.7 (13.0)	0.4 (9.9)	3.9 (16.9)	−0.8 (14.8)	−1.2 (−5.5, 3.2)	0.604	7.3 (17.0)	−3.5 (11.4)	−3.0 (−7.3, 1.3)	0.177
Fatigue score ^d^										
2 weeks of intervention	1.0 (14.6)	1.6 (8.9)	0.2 (15.8)	−1.0 (9.2)	−2.8 (−6.3, 0.6)	0.108	2.4 (16.7)	−2.8 (10.7)	−3.8 (−7.4, −0.2)	0.038
4 weeks of intervention ^c^	1.0 (14.6)	0.9 (8.9)	0.1 (16.0)	−1.5 (12.8)	−2.6 (−6.4, 1.3)	0.194	1.6 (15.9)	−1.5 (9.7)	1.5 (−4.4, 7.4)	0.617
Well-being score ^e^										
2 weeks of intervention	49.0 (7.0)	−1.9 (8.3)	49.0 (9.4)	0.8 (4.4)	2.8 (0.5, 5.1)	0.018	47.8 (9.3)	1.7 (5.4)	3.4 (1.2, 5.7)	0.003
4 weeks of intervention ^c^	49.0 (7.9)	0.2 (6.6)	49.2 (9.4)	1.8 (7.8)	1.7 (−0.8, 4.3)	0.180	48.2 (9.0)	2.4 (7.0)	2.0 (−0.5, 4.5)	0.124

Note: POMS = Profile of Mood States questionnaire. ^a^ Mean differences, 95% CI, and *p*-values determined using a mixed effects regression model adjusted for baseline scores and with the two randomisation clusters as random effects. ^b^ Higher score means higher mood disturbance overall (worse mood) (minimim possible score = −20, maximum = 100). ^c^ One participant in each of the vitamin C and kiwifruit groups did not have data at 4 weeks of intervention. ^d^ Higher multi-dimensional fatigue score means higher fatigue (minimum possible score = −24, maximum = 96). ^e^ Higher well-being score means higher well-being (minimum possible score = 14, maximum = 70).

**Table 6:** Effect of Vitamin C tablet or Kiwifruit on mood disturbance, fatigue, and well-being after 2 and 4 weeks of intervention, compared to week 2 of baseline (end of lead-in), for those with plasma vitamin C below saturation < 60 μmol/L before intervention (*n* = 128) and those with plasma vitamin C below 40 μmol/L before the intervention, as per protocol (*n* = 92).

	Plasma Vitamin C Below Saturation (*n* = 128)				Per Protocol (*n* = 92)			
	Vitamin C Tablet Group (*n* = 40)		Kiwifruit Group (*n* = 43)		Vitamin C Tablet Group (*n* = 29)		Kiwifruit Group (*n* = 27)	
	Mean Difference in Change (95% CI) Compared to Placebo ^a^	*p*-Value	Mean Difference in Change (95% CI) Compared to Placebo ^a^	*p*-Value	Mean Difference in Change (95% CI) Compared to Placebo ^a^	*p*-Value	Mean Difference in Change (95% CI) Compared to Placebo ^a^	*p*-Value
POMS total score ^b^								
2 weeks of intervention	−4.2 (−8.5, 0.2)	0.060	−3.9 (−8.5, 0.6)	0.087	−7.9 (−13.2, −2.6)	0.003	−4.5 (−10.3, 1.2)	0.121
4 weeks of intervention ^c^	−3.5 (−7.8, 0.7)	0.105	−2.8 (−7.3, 1.6)	0.212	−3.6 (−8.4, 1.2)	0.137	−3.6 (−8.7, 1.6)	0.174
Fatigue score ^c^								
2 weeks of intervention	−3.7 (−7.2, −0.2)	0.036	−4.4 (−8.0, −0.8)	0.017	−5.9 (−9.9, −1.8)	0.005	−4.1 (−8.5, 0.4)	0.073
4 weeks of intervention ^c^	−4.7 (−8.5, −0.9)	0.016	−1.4 (−5.3, 2.6)	0.505	−4.4 (−8.9, 0.1)	0.057	−0.7 (−5.5, 4.2)	0.790
Well-being score ^d^								
2 weeks of intervention	2.5 (0.01, 5.0)	0.049	4.8 (2.2, 7.4)	< 0.001	3.0 (0.0, 6.0)	0.047	4.1 (0.7, 7.6)	0.017
4 weeks of intervention ^c^	2.3 (−0.3, 4.9)	0.077	2.7 (−0.01, 5.4)	0.051	2.2 (−0.7, 5.1)	0.143	2.6 (−0.6, 5.7)	0.112

Note: POMS = Profile of Mood States questionnaire; ^a^ Mean differences, 95% CI, and p-values determined using a mixed effects regression model adjusted for baseline scores, age, ethnicity, years of university study, and plasma vitamin C levels before the intervention, and with the two randomisation clusters as random effects. ^b^ Higher score means higher mood disturbance overall (worse mood) (minimim possible score = −20, maximum = 100). ^c^ Higher multi-dimensional fatigue score means higher fatigue (minimum possible score = −24, maximum = 96). ^d^ Higher well-being score means higher well-being (minimum possible score = 14, maximum = 70).

## Supplementary Materials

The following are available online at https://www.mdpi.com/2072-6643/12/9/2898/s1, Figure S1: Below saturation vitamin C sample (*n* = 128) changes in fortnightly measures of vitality (total mood disturbance, fatigue, and well-being) over the study period, Figure S2: Per protocol (PP) sample (*n* = 92) changes in fortnightly measures of vitality (total mood disturbance, fatigue, and well-being) over the study period, Table S1: Available cases sample (*n* = 167) raw mean scores (and standard deviations) of the primary outcome measures and secondary covariates overall and by condition, Table S2: Available cases sample (*n* = 167) macronutrient diet record data (kilojoules of intake daily) for the placebo, vitamin C, and kiwifruit conditions, Table S3: Below saturation vitamin C sample (*n* = 128) participant characteristics overall and by condition, Table S4: Below saturation vitamin C sample (*n* = 128) plasma vitamin C concentrations (µmol/L) by condition during the study period, Table S5: Below saturation vitamin C sample (*n* = 128) raw mean scores (and standard deviations) of primary outcome measures and secondary covariates overall and by condition, Table S6: Below saturation vitamin C sample (*n* = 128) macronutrient diet record data for the placebo, vitamin C, and kiwifruit conditions, Table S7: Per protocol sample (*n* = 92) participant characteristics overall and by condition, Table S8: Per protocol sample (*n* = 92) plasma vitamin C concentrations (µmol/L) by condition during the study period, Table S9: Per protocol sample (*n* = 92) raw mean scores (and standard deviations) of the primary outcome measures and secondary covariates overall and by condition, Table S10: Per protocol sample (*n* = 92) macronutrient diet record data for the placebo, vitamin C, and kiwifruit conditions, Table S11: Discontinued participants (cumulative percentage (*n*)), and Table S12: Sensitivity analysis using pattern mixture models.

## References

[1] Blanchflower D.G., Oswald A.J., Stewart-Brown S. (2012). Is psychological well-being linked to the consumption of fruit and vegetables?. Soc. Indic. Res..

[2] Piqueras J.A., Kuhne W., Vera-Villarroel P., Van Straten A., Cuijpers P. (2011). Happiness and health behaviours in Chilean college students: A cross-sectional survey. BMC Public Health.

[3] White B.A., Horwath C.C., Conner T.S. (2013). Many apples a day keep the blues away-daily experiences of negative and positive affect and food consumption in young adults. Br. J. Health Psychol..

[4] Crichton G.E., Bryan J., Hodgson J.M., Murphy K.J. (2013). Mediterranean diet adherence and self-reported psychological functioning in an Australian sample. Appetite.

[5] Głąbska D., Guzek D., Groele B., Gutkowska K. (2020). Fruit and vegetable intake and mental health in adults: A systematic review. Nutrients.

[6] Block G., Norkus E., Hudes M., Mandel S., Helzlsouer K. (2001). Which plasma antioxidants are most related to fruit and vegetable consumption?. Am. J. Epidemiol..

[7] Granger M., Eck P. (2018). Dietary Vitamin C in human health. Adv. Food Nutr. Res..

[8] Levine M., Rumsey S.C., Wang Y., Park J., Kwon O., Amano N. (1997). In situ kinetics: An approach to recommended intake of vitamin C. Methods Enzymol..

[9] Rumsey S., Levine M. (1998). Absorption, transport, and disposition of ascorbic acid in humans. Nutr. Biochem..

[10] Levine M., Wang Y., Padayatty S.J., Morrow J. (2001). A new recommended dietary allowance of vitamin C for healthy young women. Proc. Natl. Acad. Sci. USA.

[11] Hasselholt S., Tveden-Nyborg P., Lykkesfeldt J. (2015). Distribution of vitamin C is tissue specific with early saturation of the brain and adrenal glands following differential oral dose regimens in guinea pigs. Br. J. Nutr..

[12] Meredith M.E., May J.M. (2013). Regulation of embryonic neurotransmitter and tyrosine hydroxylase protein levels by ascorbic acid. Brain Res..

[13] Prigge S.T., Mains R.E., Eipper B.A., Amzel L.M. (2000). New insights into copper monooxygenases and peptide amidation: Structure, mechanism and function. Cell. Mol. Life Sci..

[14] Levine M. (1986). Ascorbic acid specifically enhances dopamine beta-monooxygenase activity in resting and stimulated chromaffin cells. J. Biol. Chem..

[15] Pihlajaniemi T., Myllyla R., Kivirikko K.I. (1991). Prolyl 4-hydroxylase and its role in collagen synthesis. J. Hepatol..

[16] Levine M., Conry-Cantilena C., Wang Y., Welch R.W., Washko P.W., Dhariwal K.R., Park J.B., Lazarev A., Graumlich J.F., King J. (1996). Vitamin C pharmacokinetics in healthy volunteers: Evidence for a recommended dietary allowance. Proc. Natl. Acad. Sci. USA.

[17] Carr A.C., Frei B. (1999). Toward a new recommended dietary allowance for vitamin C based on antioxidant and health effects in humans. Am. Soc. Clin. Nutr..

[18] Agarwal A., Shaharyar A., Kumar A., Bhat M.S., Mishra M. (2015). Scurvy in pediatric age group—A disease often forgotten?. J. Clin. Orthop. Trauma.

[19] Harrison F.E., May J.M. (2009). Vitamin C function in the brain: Vital role of the ascorbate transporter SVCT2. Free Radic. Biol. Med..

[20] Kinsman R.A., Hood J. (1971). Some behavioral effects of ascorbic acid deficiency. Am. J. Clin. Nutr..

[21] Lykkesfeldt J., Poulsen H.E. (2010). Is vitamin C supplementation beneficial? Lessons learned from randomised controlled trials. Br. J. Nutr..

[22] Léger D. (2008). Scurvy. Can. Fam. Physician.

[23] Cheraskin E., Ringsdorf W.M.J., Medford F.H. (1976). Daily vitamin C consumption and fatigability. J. Am. Geriatr. Soc..

[24] Johnston C.S., Barkyoumb G.M., Schumacher S.S. (2014). Vitamin C supplementation slightly improves physical activity levels and reduces cold incidence in men with marginal vitamin C status: A randomized controlled trial. Nutrients.

[25] Conner T.S., Brookie K.L., Carr A.C., Mainvil L.A., Vissers M.C. (2017). Let them eat fruit! The effect of fruit and vegetable consumption on psychological well-being in young adults: A randomized controlled trial. PLoS ONE.

[26] Mujcic R., Oswald A.J. (2016). Evolution of well-being and happiness after increases in consumption of fruit and vegetables. Am. J. Public Health.

[27] Rooney C., McKinley M.C., Woodside J.V. (2013). The potential role of fruit and vegetables in aspects of psychological well-being: A review of the literature and future directions. Proc. Nutr. Soc..

[28] Jacka F.N., Pasco J.A., Mykletun A., Williams L.J., Hodge A.M., O’Reilly S.L., Nicholson G.C., Kotowicz M.A., Berk M. (2010). Association of western and traditional diets with depression and anxiety in women. Am. J. Psychiatry.

[29] Choi J.E., Ainsworth B.E. (2016). Associations of food consumption, serum vitamins and metabolic syndrome risk with physical activity level in middle-aged adults: The National Health and Nutrition Examination Survey (NHANES) 2005–2006. Public Health Nutr..

[30] Paschalis V., Theodorou A.A., Kyparos A., Dipla K., Zafeiridis A., Panayiotou G., Vrabas I.S., Nikolaidis M.G. (2016). Low vitamin C values are linked with decreased physical performance and increased oxidative stress: Reversal by vitamin C supplementation. Eur. J. Nutr..

[31] Harding A.H., Wareham N.J., Bingham S.A., Khaw K., Luben R., Welch A., Forouhi N.G. (2008). Plasma vitamin C level, fruit and vegetable consumption, and the risk of new-onset type 2 diabetes mellitus: The European prospective investigation of cancer—Norfolk prospective study. Arch. Intern. Med..

[32] Carr A.C., Bozonet S.M., Pullar J.M., Vissers M.C. (2013). Mood improvement in young adult males following supplementation with gold kiwifruit, a high-vitamin C food. J. Nutr. Sci..

[33] Nishiyama I., Yamashita Y., Yamanaka M., Shimohashi A., Fukuda T., Oota T. (2004). Varietal difference in vitamin C content in the fruit of kiwifruit and other actinidia species. J. Agric. Food Chem..

[34] Griskevicius V., Delton A.W., Robertson T.E., Tybur J.M. (2011). Environmental contingency in life history strategies: The influence of mortality and socioeconomic status on reproductive timing. J. Personal. Soc. Psychol..

[35] McNair D.M., Heuchert J.W.P. (2005). Profiles of Mood States—Technical Update.

[36] Stein K.D., Jacobsen P.B., Blanchard C.M., Thors C. (2004). Further Validation of the Multidimensional Fatigue Symptom Inventory-Short Form. J. Pain Symptom Manag..

[37] Tennant R., Hiller L., Fishwick R., Platt S., Joseph S., Weich S., Parkinson J., Secker J., Stewart-Brown S. (2007). The Warwick-Edinburgh Mental Well-being Scale (WEMWBS): Development and UK validation. Health Qual. Life Outcomes.

[38] Pullar J.M., Bayer S., Carr A.C. (2018). Appropriate handling, processing and analysis of blood samples is essential to avoid oxidation of vitamin C to dehydroascorbic acid. Antioxidants.

[39] Carr A.C., Bozonet S.M., Pullar J.M., Simcock J.W., Vissers M.C. (2013). A randomized steady-state bioavailability study of synthetic versus natural (kiwifruit-derived) vitamin C. Nutrients.

[40] Kruse R.L., Alper B.S., Reust C., Stevermer J.J., Shannon S., Williams R.H. (2002). Intention-to-treat analysis: Who is in? Who is out?. J. Fam. Pract..

[41] Jakobsen J.C., Gluud C., Wetterslev J., Winkel P. (2017). When and how should multiple imputation be used for handling missing data in randomised clinical trials–a practical guide with flowcharts. BMC Med Res. Methodol..

[42] Dettori J.R., Norvell D.C., Chapman J.R. (2018). The sin of missing data: Is all forgiven by way of imputation?. Glob. Spine J..

[43] National Research Council (2010). The prevention and treatment of missing data in clinical trials. Panel on Handling Missing Data in Clinical Trials.

[44] Carr A.C., Vissers M.C. (2013). Synthetic or food-derived vitamin C-are they equally bioavailable?. Nutrients.

[45] Richardson D.P., Ansell J., Drummond L.N. (2018). The nutritional and health attributes of kiwifruit: A review. Eur. J. Nutr..

[46] U.S. Department of Agriculture (2018). National Nutrient Database for Standard Reference Legacy Release.

[47] Dash S., Clarke G., Berk M., Jacka F.N. (2015). The gut microbiome and diet in psychiatry: Focus on depression. Curr. Opin. Psychiatry.

[48] Scott K.P., Duncan S.H., Flint H.J. (2008). Dietary fibre and the gut microbiota. Nutr. Bull..

[49] Tap J., Furet J.-P., Bensaada M., Philippe C., Roth H., Rabot S., Lakhdari O., Lombard V., Henrissat E., Corthier G. (2015). Gut microbiota richness promotes its stability upon increased dietary fibre intake in healthy adults. Environ. Microbiol..

[50] Morris M.S., Fava M., Jacques P.F., Selhub J., Rosenberg I.H. (2003). Depression and folate status in the US population. Psychother. Psychosom..

[51] Torres S.J., Nowson C.A., Worsley A. (2008). Dietary electrolytes are related to mood. Br. J. Nutr..

[52] Staudacher H.M., Irving P.M., Lomer M.C., Whelan K. (2017). The challenges of control groups, placebos and blinding in clinical trials of dietary interventions. Proc. Nutr. Soc..

[53] Adan R.A., van der Beek E.M., Buitelaar J.K., Cryan J.F., Hebebrand J., Higgs S., Schellekens H., Dickson S.L. (2019). Nutritional psychiatry: Towards improving mental health by what you eat. Eur. Neuropsychopharmacol..

[54] Michels A.J., Frei B. (2013). Myths, artifacts, and fatal flaws: Identifying limitations and opportunities in vitamin C research. Nutrients.

[55] Dunn W.A., Rettura G., Seifter E., Englard S. (1984). Carnitine biosynthesis from gamma-butyrobetaine and from exogenous protein-bound 6-N-trimethyl-L-lysine by the perfused guinea pig liver. Effect of ascorbate deficiency on the in situ activity of gamma-butyrobetaine hydroxylase. J. Biol. Chem..

[56] Rebouche C.J. (1991). Ascorbic acid and carnitine biosynthesis. Am. J. Clin. Nutr..

[57] Nelson P.J., Pruitt R.E., Henderson L.L., Jenness R., Henderson L.M. (1981). Effect of ascorbic acid deficiency on the in vivo synthesis of carnitine. Biochim. Biophys. Acta.

[58] Noland R.C., Koves T.R., Seiler S.E., Lum H., Lust R.M., Ilkayeva O., Stevens R.D., Hegardt F.G., Muoio D.M. (2009). Carnitine insufficiency caused by aging and overnutrition compromises mitochondrial performance and metabolic control. J. Biol. Chem..

[59] Rush R.A., Geffen L.B. (1980). Dopamine beta-hydroxylase in health and disease. Crit. Rev. Clin. Lab. Sci..

[60] May J.M., Qu Z.C., Nazarewicz R., Dikalov S. (2013). Ascorbic acid efficiently enhances neuronal synthesis of norepinephrine from dopamine. Brain Res. Bull..

[61] Hoehn S.K., Kanfer J.N. (1980). Effects of chronic ascorbic acid deficiency on guinea pig lysosomal hydrolase activities. J. Nutr..

[62] Deana R., Bharaj B.S., Verjee Z.H., Galzigna L. (1975). Changes relevant to catecholamine metabolism in liver and brain of ascorbic acid deficient guinea-pigs. Int. J. Vitam. Nutr. Res..

[63] Bornstein S.R., Yoshida-Hiroi M., Sotiriou S., Levine M., Hartwig H.G., Nussbaum R.L., Eisenhofer G. (2003). Impaired adrenal catecholamine system function in mice with deficiency of the ascorbic acid transporter (SVCT2). FASEB J..

[64] May J.M., Qu Z.C., Meredith M.E. (2012). Mechanisms of ascorbic acid stimulation of norepinephrine synthesis in neuronal cells. Biochem. Biophys. Res. Commun..

[65] Kringelbach M.L., Berridge K.C. (2009). Toward a functional neuroanatomy of pleasure and happiness. Trends Cogn. Sci..

[66] Englard S., Seifter S. (1986). The biochemical functions of ascorbic acid. Annu. Rev. Nutr..

[67] Eipper B.A., Mains R.E. (1991). The role of ascorbate in the biosynthesis of neuroendocrine peptides. Am. J. Clin. Nutr..

[68] Kukucka M.A., Misra H.P. (1992). HPLC determination of an oxytocin-like peptide produced by isolated guinea pig Leydig cells: Stimulation by ascorbate. Arch. Androl..

[69] Sheldrick E.L., Flint A.P. (1989). Post-translational processing of oxytocin-neurophysin prohormone in the ovine corpus luteum: Activity of peptidyl glycine alpha-amidating mono-oxygenase and concentrations of its cofactor, ascorbic acid. J. Endocrinol..

[70] Ishak W.W., Kahloon M., Fakhry H. (2011). Oxytocin role in enhancing well-being: A literature review. J. Affect. Disord..

[71] Luck M.R., Jungclas B. (1987). Catecholamines and ascorbic acid as stimulators of bovine ovarian oxytocin secretion. J. Endocrinol..

