# Correction: Conner et al. KiwiC for Vitality: Results of a Placebo-Controlled Trial Testing the Effects of Kiwifruit or Vitamin C Tablets on Vitality in Adults with Low Vitamin C Levels. *Nutrients* 2020, *12*, 2898

**DOI:** 10.3390/nu14194063

**Published:** 2022-09-30

**Authors:** Tamlin S. Conner, Benjamin D. Fletcher, Jillian J. Haszard, Juliet M. Pullar, Emma Spencer, Louise A. Mainvil, Margreet C. M. Vissers

**Affiliations:** 1 Department of Psychology, University of Otago, Dunedin 9054, New Zealand; 2 Centre for Free Radical Research, Department of Pathology and Biomedical Science, University of Otago, Christchurch 8140, New Zealand; 3 Department of Pathology and Biomedical Science, University of Otago, Christchurch 8140, New Zealand; 4 Department of Human Nutrition, University of Otago, Dunedin 9054, New Zealand

## Title Correction

In the original publication title [1], we referred to the study as a randomized placebo-controlled trial. Due to compromised randomization, we have amended the title to remove the word randomized. The corrected article title should be: KiwiC for Vitality: Results of a Placebo-Controlled Trial Testing the Effects of Kiwifruit or Vitamin C Tablets on Vitality in Adults with Low Vitamin C Levels.

## Addition of an Author

Dr. Jillian J. Haszard was not included as an author in the original publication. J.J.H. joined the team after the original publication to lead the statistical reanalysis. Thus, we have added authorship in the corrected paper. 

New Authorship:

Tamlin S. Conner ^1,^*, Benjamin D. Fletcher ^1^, Jillian J. Haszard ^2^, Juliet M. Pullar ^2^, Emma Spencer ^3^, Louise A. Mainvil ^4^ and Margreet C. M. Vissers ^2^

New Authorship Statement:

**Author Contributions:** Conceptualization, T.S.C. and M.C.M.V.; methodology, T.S.C. and M.C.M.V.; statistical analyses, J.J.H., T.S.C., B.D.F., and M.C.M.V.; investigation, T.S.C., B.D.F., J.M.P., and E.S.; resources, T.S.C., L.A.M., and M.C.M.V.; writing—original draft preparation, T.S.C., B.D.F., and M.C.M.V.; writing—review and editing, T.S.C., B.D.F., J.M.P., and M.C.M.V.; visualization, J.J.H. and M.C.M.V.; supervision, T.S.C. and M.C.M.V.; funding acquisition, T.S.C. and M.C.M.V. All authors have read and agreed to the published version of the manuscript.

## Error in Abstract and Table

In the original publication, the statistical analyses did not take into account possible dependency in randomisation groupings. Therefore, analyses were re-run using mixed- effects modelling with two randomization clusters as random effects to account for any correlated errors. The corrected results are presented in the Abstract, Table 5 and Table 6 presented below. Few substantive changes were observed from the original findings aside from minor changes to effect sizes and *p*-values. 

Corrected Abstract: 

**Abstract:** Consumption of vitamin C-rich fruits and vegetables has been associated with greater feelings of vitality. However, these associations have rarely been tested in experimental trials. The aim of the current study was to test the effects of eating a vitamin C-rich food (kiwifruit) on subjective vitality and whether effects are driven by vitamin C. Young adults (*n* = 167, 61.1% female, aged 18–35 years) with plasma vitamin C < 40 µmol/L were allocated to three intervention conditions: kiwifruit (2 SunGold™ kiwifruit/day), vitamin C (250 mg tablet/day), placebo (1 tablet/day). The trial consisted of a two-week lead-in, four-week intervention, and two-week washout. Plasma vitamin C and vitality questionnaires (total mood disturbance, fatigue, and well-being) were measured fortnightly. Self-reported sleep quality and physical activity were measured every second day through smartphone surveys. Nutritional confounds were assessed using a three-day food diary during each study phase. Plasma vitamin C reached saturation levels within two weeks for the kiwifruit and vitamin C groups. Participants consuming kiwifruit showed a trend of improvement in mood disturbance, significantly decreased fatigue, and significantly improved well-being after two weeks of the intervention. Improvements in well-being remained elevated through washout. Consumption of vitamin C tablets alone was associated with improved well-being after two weeks, and additionally improved mood and fatigue for participants with consistently low vitamin C levels during lead-in. Diet records showed that participants consuming kiwifruit reduced their fat intake during the intervention period. Intervention effects remained significant when adjusting for condition allocation groupings, age, and ethnicity, and were not explained by sleep quality, physical activity, BMI, or other dietary patterns, including fat intake. There were no changes in plasma vitamin C status or vitality in the placebo group. Whole-food consumption of kiwifruit was associated with improved subjective vitality in adults with low vitamin C status. Similar, but not identical changes were found for vitamin C tablets, suggesting that additional properties of kiwifruit may contribute to improved vitality.

Corrected Table 5 and newly added Table 6: 

**Table 5:** Effect of Vitamin C tablet or Kiwifruit on mood disturbance, fatigue, and well-being after 2 and 4 weeks of intervention, compared to week 2 of baseline (end of Lead in) (*n* = 161).

	Placebo Group (*n* = 52)		Vitamin C Tablet Group (*n* = 53)				Kiwifruit Group (*n* = 56)			
	Baseline Mean (SD)	Mean Change from Baseline (SD)	Baseline Mean (SD)	Mean Change from Baseline (SD)	Mean Difference in Change (95% CI) Compared to Placebo ^a^	*p*-Value	Baseline Mean (SD)	Mean Change from Baseline (SD)	Mean Difference in Change (95% CI) Compared to Placebo ^a^	*p*- Value
POMS total score ^b^										
2 weeks of intervention	3.7 (13.0)	1.8 (13.1)	3.9 (16.7)	−1.1 (10.1)	−3.0 (−6.9, 0.8)	0.121	8.6 (19.4)	−3.7 (8.9)	−4.0 (−8.1, 0.2)	0.062
4 weeks of intervention ^c^	3.7 (13.0)	0.4 (9.9)	3.9 (16.9)	−0.8 (14.8)	−1.2 (−5.5, 3.2)	0.604	7.3 (17.0)	−3.5 (11.4)	−3.0 (−7.3, 1.3)	0.177
Fatigue score ^d^										
2 weeks of intervention	1.0 (14.6)	1.6 (8.9)	0.2 (15.8)	−1.0 (9.2)	−2.8 (−6.3, 0.6)	0.108	2.4 (16.7)	−2.8 (10.7)	−3.8 (−7.4, −0.2)	0.038
4 weeks of intervention ^c^	1.0 (14.6)	0.9 (8.9)	0.1 (16.0)	−1.5 (12.8)	−2.6 (−6.4, 1.3)	0.194	1.6 (15.9)	−1.5 (9.7)	1.5 (−4.4, 7.4)	0.617
Well-being score ^e^										
2 weeks of intervention	49.0 (7.0)	−1.9 (8.3)	49.0 (9.4)	0.8 (4.4)	2.8 (0.5, 5.1)	0.018	47.8 (9.3)	1.7 (5.4)	3.4 (1.2, 5.7)	0.003
4 weeks of intervention ^c^	49.0 (7.9)	0.2 (6.6)	49.2 (9.4)	1.8 (7.8)	1.7 (−0.8, 4.3)	0.180	48.2 (9.0)	2.4 (7.0)	2.0 (−0.5, 4.5)	0.124

Note: POMS = Profile of Mood States questionnaire. ^a^ Mean differences, 95% CI, and *p*-values determined using a mixed effects regression model adjusted for baseline scores and with the two randomisation clusters as random effects. ^b^ Higher score means higher mood disturbance overall (worse mood) (minimim possible score = −20, maximum = 100). ^c^ One participant in each of the vitamin C and kiwifruit groups did not have data at 4 weeks of intervention. ^d^ Higher multi-dimensional fatigue score means higher fatigue (minimum possible score = −24, maximum = 96). ^e^ Higher well-being score means higher well-being (minimum possible score = 14, maximum = 70).

**Table 6:** Effect of Vitamin C tablet or Kiwifruit on mood disturbance, fatigue, and well-being after 2 and 4 weeks of intervention, compared to week 2 of baseline (end of lead-in), for those with plasma vitamin C below saturation < 60 μmol/L before intervention (*n* = 128) and those with plasma vitamin C below 40 μmol/L before the intervention, as per protocol (*n* = 92).

	Plasma Vitamin C Below Saturation (*n* = 128)				Per Protocol (*n* = 92)			
	Vitamin C Tablet Group (*n* = 40)		Kiwifruit Group (*n* = 43)		Vitamin C Tablet Group (*n* = 29)		Kiwifruit Group (*n* = 27)	
	Mean Difference in Change (95% CI) Compared to Placebo ^a^	*p*-Value	Mean Difference in Change (95% CI) Compared to Placebo ^a^	*p*-Value	Mean Difference in Change (95% CI) Compared to Placebo ^a^	*p*-Value	Mean Difference in Change (95% CI) Compared to Placebo ^a^	*p*-Value
POMS total score ^b^								
2 weeks of intervention	−4.2 (−8.5, 0.2)	0.060	−3.9 (−8.5, 0.6)	0.087	−7.9 (−13.2, −2.6)	0.003	−4.5 (−10.3, 1.2)	0.121
4 weeks of intervention ^c^	−3.5 (−7.8, 0.7)	0.105	−2.8 (−7.3, 1.6)	0.212	−3.6 (−8.4, 1.2)	0.137	−3.6 (−8.7, 1.6)	0.174
Fatigue score ^c^								
2 weeks of intervention	−3.7 (−7.2, −0.2)	0.036	−4.4 (−8.0, −0.8)	0.017	−5.9 (−9.9, −1.8)	0.005	−4.1 (−8.5, 0.4)	0.073
4 weeks of intervention ^c^	−4.7 (−8.5, −0.9)	0.016	−1.4 (−5.3, 2.6)	0.505	−4.4 (−8.9, 0.1)	0.057	−0.7 (−5.5, 4.2)	0.790
Well-being score ^d^								
2 weeks of intervention	2.5 (0.01, 5.0)	0.049	4.8 (2.2, 7.4)	< 0.001	3.0 (0.0, 6.0)	0.047	4.1 (0.7, 7.6)	0.017
4 weeks of intervention ^c^	2.3 (−0.3, 4.9)	0.077	2.7 (−0.01, 5.4)	0.051	2.2 (−0.7, 5.1)	0.143	2.6 (−0.6, 5.7)	0.112

Note: POMS = Profile of Mood States questionnaire; ^a^ Mean differences, 95% CI, and *p*-values determined using a mixed effects regression model adjusted for baseline scores, age, ethnicity, years of university study, and plasma vitamin C levels before the intervention, and with the two randomisation clusters as random effects. ^b^ Higher score means higher mood disturbance overall (worse mood) (minimim possible score = −20, maximum = 100). ^c^ Higher multi-dimensional fatigue score means higher fatigue (minimum possible score = −24, maximum = 96). ^d^ Higher well-being score means higher well-being (minimum possible score = 14, maximum = 70).

## Error in Figure

In the original publication, Figure 4 presented within-group comparisons of the primary outcomes over time rather than between-group comparisons, as required by CONSORT guidelines. The corrected Figure 4 shows below.

## Text Correction

The original publication lacked detail in the randomization procedure and did not clarify that there was non-random allocation in the study implementation. This stemmed from allocating the first two weeks of participants to the kiwifruit condition due to circumstances outside our control (late arrival of tablets from overseas). The correct information appears below and in Section 2.3 of the corrected manuscript. We have also included Supplementary Figure S1 to reflect the full condition allocation schedule.

**2.3. Condition Allocation**

Our intention was to avoid participants being exposed to the other conditions during allocation of the intervention (e.g., participants seeing others receiving kiwifruit when they received tablets or vice versa). This was achieved by group randomisation based on scheduled clinic, either on separate days or across days, to ensure that everyone attending the same clinic would receive either kiwifruit or tablets. Participants receiving tablets at a given clinic were typically a random mix of the active and placebo groups. The allocation of clinics was determined by the lead author using a random number generator. As allocation occurred after participants were already enrolled into their clinic day (usually Mon–Thurs), the clinical study co-ordinator and prospective participants were unaware of their allocation when booking clinic appointments and did not learn of their allocation until they attended the clinic. A delay in the delivery of tablets meant that clinics in the first and second weeks were non-randomly allocated to the kiwifruit condition, and those in the third and fourth weeks, to the tablet conditions (randomised to placebo or vitamin C), with the remaining clinics randomised as intended. A randomisation schedule is shown in Supplementary Figure S1. Randomisation groups were accounted for in the statistical analyses (see Statistical Analyses, below). Tablets were bottled and labelled by the lead author (TSC) with the label including only the participant’s first and last name and tablet instructions to “chew one tablet daily, store in a dark dry place, and return bottle and unused tablets on next visit”. All research assistants and participants were double- blinded to their tablet condition. It was not possible to blind research assistants or participants to the kiwifruit condition. The separation of allocation clinics, however, meant that these participants were unaware of the nature of the other treatment conditions. Condition information was kept in an electronic password-protected document by the lead author (TSC) and unblinded following data collection and entry.

The authors apologize for any inconvenience caused and state that the primary scientific conclusions are unaffected. This correction was approved by the Academic Editor. The original publication has also been updated.

## Figures and Tables

**Figure 4:**
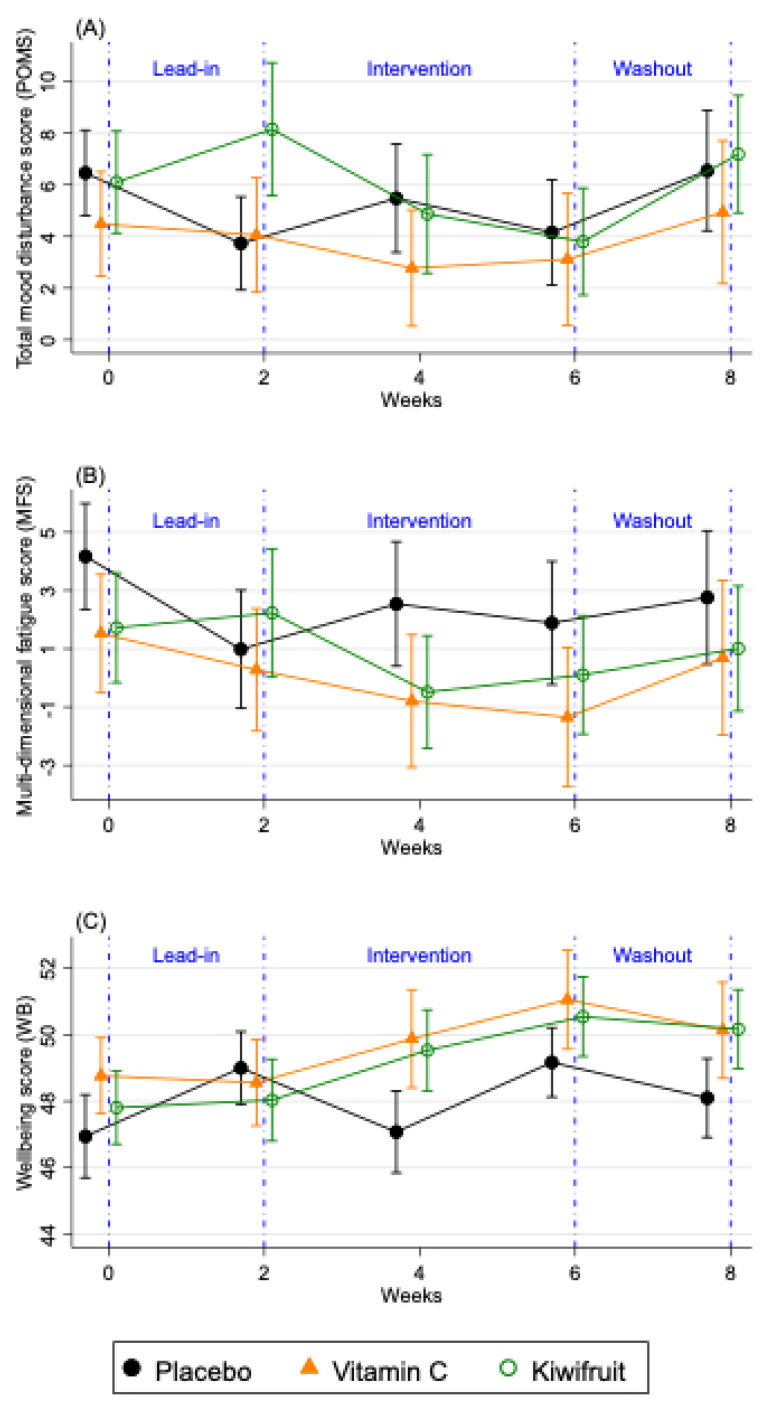
Changes in fortnightly (**A**) total mood disturbance scores (POMS), (**B**) multidimensional fatigue score (MFS), and (**C**) well-being (WB) over the study period for the total sample (*n* = 167). Results are presented as means ± SE for participants allocated to placebo tablet (black lines), vitamin C tablet (orange lines) and kiwifruit (green lines) conditions. Lead-in Week 2 served as baseline, which was compared against Week 4 and Week 6 of the intervention.

## References

[1] Conner T.S., Fletcher B.D., Haszard J.J., Pullar J.M., Spencer E., Mainvil L.A., Vissers M.C.M. (2020). KiwiC for Vitality: Results of a Placebo-Controlled Trial Testing the Effects of Kiwifruit or Vitamin C Tablets on Vitality in Adults with Low Vitamin C Levels. Nutrients.

