# Supplementary material for: KiwiC for Vitality: Results of a Placebo-Controlled Trial Testing the Effects of Kiwifruit or Vitamin C Tablets on Vitality in Adults with Low Vitamin C Levels
Source: Nutrients. 2020 Sep 22;12(9):2898. doi: 10.3390/nu12092898 (PMC7551849; doi:10.3390/nu12092898)
Supplement: Supplementary file 1 [file nutrients-12-02898-s001.pdf]

ONLINE SUPPLEMENTARY MATERIAL

KiwiC for Vitality: Results of a randomized placebo-controlled trial testing the effects of kiwifruit or vitamin C tablets on vitality in adults with low vitamin C levels

TS Conner, BD Fletcher, JM Pullar, E Spencer, LA Mainvil, MCM Vissers

CONTENTS

Available Cases Analysis Results (N = 167)

Below Saturation Vitamin C Analysis Results (N = 128)

Per Protocol Analysis Results (N = 92)

Sensitivity Analysis using Pattern Mixture Models

Supplemental Figure 1. Below saturation vitamin C sample (*n* = 128) changes in fortnightly measures of vitality (total mood disturbance, fatigue and well-being) over the study period.

Supplemental Figure 2. Per protocol (PP) sample (*n* = 92) changes in fortnightly measures of vitality (total mood disturbance, fatigue, and well-being) over the study period.

Supplemental Table 1. Available cases sample (*n* = 167) raw mean scores (and standard deviations) of the primary outcome measures and secondary covariates overall and by condition

Supplemental Table 2. Available cases sample (*n* = 167) macronutrient diet record data (kilojoules of intake daily) for the placebo, vitamin C, and kiwifruit conditions

Supplemental Table 3. Below saturation vitamin C sample (*n* = 128) participant characteristics overall and by condition

Supplemental Table 4. Below saturation vitamin C sample (*n* = 128) plasma vitamin C concentrations (µmol/L) by condition during the study period

Supplemental Table 5. Below saturation vitamin C sample (*n* = 128) raw mean scores (and standard deviations) of primary outcome measures and secondary covariates overall and by condition

Supplemental Table 6. Below saturation vitamin C sample (*n* = 128) macronutrient diet record data for the placebo, vitamin C, and kiwifruit conditions

Supplemental Table 7. Per protocol sample (*n* = 92) participant characteristics overall and by condition

Supplemental Table 8. Per protocol sample (*n* = 92) plasma vitamin C concentrations (µmol/L) by condition during the study period

Supplemental Table 9. Per protocol sample (*n* = 92) raw mean scores (and standard deviations) of the primary outcome measures and secondary covariates overall and by condition

Supplemental Table 10. Per protocol sample (*n* = 92) macronutrient diet record data for the placebo, vitamin C, and kiwifruit conditions

Supplemental Table 11. Discontinued participants (cumulative percentage (*n*))

Supplemental Table 12. Sensitivity analysis using pattern mixture models

Available Cases Analysis Results (*n* = 167)

Supplemental Table 1. Available cases sample (*n* = 167) raw mean scores (and standard deviations) of the primary outcome measures and secondary covariates overall and by condition

	Overall		Placebo		Vitamin C		Kiwifruit	
	n	Mean (SD)	*n*	Mean (SD)	*n*	Mean (SD)	*n*	Mean (SD)
Primary Outcomes								
POMS TMD (range -20 to 100)								
Week 0 (Lead-in)	167	5.68 (14.12)	54	6.46 (12.18)	56	4.48 (15.17)	57	6.11 (14.92)
Week 2 (Lead-in)	164	5.38 (16.63)	52	3.73 (13.03)	55	4.05 (16.43)	57	8.16 (19.44)
Week 4 (Intervention)	161	4.38 (16.22)	52	5.48 (15.16)	53	2.77 (16.35)	56	4.88 (17.20) **[a]
Week 6 (Intervention)	159	3.69 (16.14)	52	4.15 (14.72)	52	3.12 (18.41)	55	3.80 (15.39) *[a]
Week 8 (Washout)	155	6.24 (17.61) *[b]**[c]	51	6.55 (16.65)	51	4.94 (19.76) †[bc]	53	7.19 (16.55) †[b]*[c]
Fatigue (range -24 to 96)								
Week 0 (Lead-in)	167	2.45 (14.24)	54	4.17 (13.34)	56	1.52 (15.19)	57	1.72 (14.20)
Week 2 (Lead-in)	164	1.18 (15.57)	52	0.98 (14.60)	55	0.28 (15.56)	57	2.23 (16.61)
Week 4 (Intervention)	161	0.39 (15.44)	52	2.54 (15.38)	53	-0.79 (16.55)	56	-0.49 (14.46) †[a]
Week 6 (Intervention)	159	0.21 (15.79)	52	1.88 (15.22)	52	-1.35 (17.19)	55	0.10 (15.05)
Week 8 (Washout)	155	1.48 (16.91) †[c]	51	2.76 (16.35)	51	0.69 (18.90) *[c]	53	1.01 (15.63)
Well-Being (range 14 to 70)								
Week 0 (Lead-in)	167	47.85 (8.65)	54	46.94 (9.18)	56	48.77 (8.55)	57	47.81 (8.28)
Week 2 (Lead-in)	164	48.52 (8.96)	52	49.00 (7.90)	55	48.56 (9.64)	57	48.04 (9.31)
Week 4 (Intervention)	161	48.85 (9.63)	52	47.08 (8.89)	53	49.88 (10.74)	56	49.53 (9.11) *[a]
Week 6 (Intervention)	159	50.26 (9.10) *[ab]	52	49.17 (7.50) *[b]	52	51.06 (10.73) †[a]	55	50.55 (8.88) *[a]
Week 8 (Washout)	155	49.48 (9.16) *[c]	51	48.10 (8.47)	51	50.14 (10.31)	53	50.17 (8.64) *[a]
Secondary Covariates								
Sleep Quantity (hours/night)								
Weeks 1-2 (Lead-in)	166	7.18 (1.04)	54	7.07 (1.11)	55	7.35 (0.86)	57	7.11 (1.11)
Weeks 3-4 (Intervention)	161	7.20 (1.03)	52	7.13 (1.07)	53	7.17 (1.04)	56	7.29 (0.98)
Weeks 5-6 (Intervention)	160	7.26 (1.06)	52	7.09 (1.03)	52	7.53 (1.11) *[e]	56	7.17 (1.01)
Weeks 7-8 (Washout)	155	7.08 (0.98) †[f]	51	6.95 (0.89)	50	7.06 (1.03) *[d]*[f]	54	7.23 (1.01)
Sleep Quality								
Weeks 1-2 (Lead-in)	166	6.54 (1.29)	54	6.21 (1.42)	55	6.80 (1.29)	57	6.61 (1.12)
Weeks 3-4 (Intervention)	161	6.61 (1.26)	52	6.53 (1.35) *[d]	53	6.78 (1.24)	56	6.54 (1.19)
Weeks 5-6 (Intervention)	160	6.78 (1.20) **[d]*[e]	52	6.65 (1.27) **[d]	52	6.92 (1.3)	56	6.76 (1.04) †[e]
Weeks 7-8 (Washout)	155	6.73 (1.27) *[d]	51	6.54 (1.35) **[d]	50	6.84 (1.26)	54	6.81 (1.21) †[e]
Physical Activity (% days active)								
Weeks 1-2 (Lead-in)	166	46.1%	54	47.5%	55	45.5%	57	45.5%
Weeks 3-4 (Intervention)	161	46.3%	52	46.9%	53	48.4%	56	43.9%
Weeks 5-6 (Intervention)	160	43.8%	52	44.7%	52	43.0%	56	43.7%
Weeks 7-8 (Washout)	155	44.6%	51	42.4%	50	48.4%	54	43.2%

Note. TMD = Total Mood Disturbance from the Profile of Mood States – Short Form Questionnaire. [a] Paired t-test comparison with Week 2 (Lead-in). [b] Paired t-test comparison with Week 4 (Intervention). [c] Paired t-test comparison with Week 6 (Intervention). [d] Paired t-test comparison with average smartphone survey responses during Weeks 1-2 (Lead-in). [e] Paired t-test comparison with average smartphone survey responses during Weeks 3-4 (Intervention). [f] Paired t-test comparison with average smartphone survey responses during Weeks 5-6 (Intervention). † $p < .10$, * $p < .05$, ** $p < .01$.

Supplemental Table 2. Available cases sample (*n* = 167) macronutrient diet record data (kilojoules of intake daily) for the placebo, vitamin C, and kiwifruit conditions

	Placebo (n = 54)		Vitamin C (n = 56)		Kiwifruit (n = 57)	
	n	Mean (SD)	*n*	Mean (SD)	*n*	Mean (SD)
Energy Intake (KJ)						
Weeks 1, Week 2 (Lead-in)	52	7818.00 (2634.58)	54	8135.51 (2739.36)	56	7717.35 (2304.98)
Week 5, Week 6 (Intervention)	52	7465.19 (3025.98)	52	8418.16 (3095.84)	55	7236.35 (2111.52)
Week 7, Week 8 (Washout)	50	6965.46 (2082.28) **[a]†[b]	49	7743.00 (2543.03) †[b]	52	7191.44 (1834.84)
Protein intake (grams)						
Week 1, Week 2 (Lead-in)	52	80.66 (32.15)	54	77.76 (42.24)	56	77.82 (34.12)
Week 5, Week 6 (Intervention)	52	76.87 (35.80)	52	86.57 (78.40)	55	77.37 (33.37)
Week 7, Week 8 (Washout)	50	70.07 (25.86) **[a]*[b]	49	78.05 (49.93)	52	73.54 (28.74)
Carbohydrate intake (grams)						
Week 1, Week 2 (Lead-in)	52	205.81 (80.78)	54	217.15 (75.42)	56	202.67 (62.17)
Week 5, Week 6 (Intervention)	52	192.93 (77.91)	52	220.97 (80.07)	55	198.06 (59.32)
Week 7, Week 8 (Washout)	50	187.24 (56.62) †[a]	49	204.38 (66.26)	52	186.32 (59.48)
Fat intake (grams)						
Week 1, Week 2 (Lead-in)	52	78.71 (30.84)	54	82.68 (37.42)	56	77.07 (30.46)
Week 5, Week 6 (Intervention)	52	77.03 (43.63)	52	84.19 (36.91)	55	67.31 (24.90) †[a]
Week 7, Week 8 (Washout)	50	68.91 (28.49) *[a]	49	78.49 (33.23)	52	73.03 (23.17)

Note. Results shown are means (standard deviations). KJ = kilojoules. [a] Paired t-test comparison with Lead-in; [b] Paired t-test comparison with Intervention. † $p < .1$, * $p < .05$, ** $p < .01$

Below Saturation Vitamin C Analysis Results (*n* = 128)

Subgroup analyses were conducted on 128 people who maintained below saturation plasma vitamin C levels (<60 μmol/L) across the two lead-in blood samples (Week 0 and Week 2). There were few differences in demographic characteristics, baseline health characteristics, or baseline vitality measures between those 128 participants included in the below saturation vitamin C analyses compared to the 39 participants excluded from these analyses (using Chi-Square statistics or t-tests, all not significant), except that included participants drank less alcohol (t(165) = -2.116, *p* = .036) and were more likely to be Asian (χ^2(1, 167) = 6.152, p = .013) than excluded participants.

Participant characteristics for this subgroup are shown in **Supplemental Table 3.** Of the 128 participants, 45 were in the placebo condition, 40 were in the vitamin C condition, and 43 were in the kiwifruit condition. The three conditions were mostly equivalent in demographic characteristics, baseline health characteristics, baseline vitality measures, and baseline vitamin C levels (all ANOVAs or Chi-Squares not significant), except that participants in the kiwifruit condition were younger (F (2,125) = 6.815, *p* = .002) than the other conditions. There were also three trends indicating that participants in the kiwifruit condition completed fewer years of study (F(2,125) = 2.920, p =.054), had lower sleep quality during the lead-in (F(2,125) = 2.631, p =.076), and were less likely to be Asian (χ^2(2,128) = 5.95, p = .051) than the other conditions. The changes in vitamin C levels for this subgroup are shown in **Supplemental Table 4.**

The changes in the vitality outcomes by condition for the below saturation vitamin C analyses (*n* = 128) are shown in **Supplemental Table 5** and **Supplemental Figure 1**. For the POMS total mood disturbance, only participants in the kiwifruit condition (**Supplemental Figure 1C**) showed significantly reduced total mood disturbance after two weeks of intervention (Study Week 4), but not at the end of the intervention (Study Week 6) or Washout (Study Week 8). Participants in the vitamin C condition showed non-significant reductions in their POMS total mood disturbance (**Supplemental Figure 1B**) and those in the placebo condition were unchanged over time (**Supplemental Figure 1A**). Participants in both the vitamin C and kiwifruit conditions showed a reduction in fatigue, either after two weeks of supplementation (**Supplemental Figure 1F**), or four weeks of supplementation (**Supplemental Figure 1E**). Fatigue scores returned to baseline levels at washout for both conditions. No changes were seen in the placebo group. For well-being, there was a significant increase in well-being after a week of kiwifruit supplementation, which stayed elevated at washout (**Supplemental Figure 1I**). Participants in the vitamin C group showed an increase in well-being after four weeks of intervention (**Supplemental Figure 1H**), which was eliminated at washout. There was no change in well-being throughout the study in the placebo group except for a trend decrease after two weeks of placebo supplementation (**Supplemental Figure 1G**).

Analysis of variance (**Table 5**, main manuscript) methods showed significant condition x time effects when comparing Study Week 2 (end of lead-in) with Study Week 4 (second week of intervention) for POMS total mood disturbance (F(2, 125) = 3.167, *p* = .046, η_p^2 = 0.048), fatigue (F(2, 125) = 3.563, *p* = .031, η_p^2 = 0.054), and well-being (F(2, 125) = 6.177, *p* = .003, η_p^2 = 0.090). When comparing Study Week 2 (end of lead-in) with Study Week 6 (end of intervention), there was a trend condition x time effect for fatigue (F(2,125) = 2.993, p =.054, η_p^2 = 0.046) but not for POMS total mood disturbance (F(2,125) = 1.919, *p* =.151, η_p^2 = 0.030) or well-being (F(2,125) = 1.995, *p* = .140, η_p^2 = 0.031). When comparing Study Week 2, Week 4, and Week 6, condition

x time effects were significant for fatigue (F(2,125) = 3.175, p = .014, η_p^2 = 0.048) and well-being (F(2,125) = 3.514, p = .008, η_p^2 = 0.053) but not for POMS total mood disturbance (F(2,125) = 1.944, p = .104, η_p^2 = 0.030).

The intervention effects in the below saturation vitamin C analyses (n = 128) were not due to differences between conditions in sleep quantity (intervention weeks 3-4, F(2,125) = 0.086, p = .918; intervention weeks 5-6, F(2,125) = 0.677, p = .510), sleep quality (intervention weeks 3-4, F(2,125) = 0.900, p = .409; intervention weeks 5-6, F(2,125) = 0.861, p = .425), physical activity (intervention weeks 3-4, F(2,125) = 0.655, p = .521; intervention weeks 5-6, F(2,125) = 0.155, p = .856), BMI at end of intervention (*Welch's F*(2,124) = 79.653, p = .932), or dietary factors such as total energy intake (KJ) (*Welch's F* (2,82.375) = 2.153, p = .123), or total protein intake (*Welch's F* (2,78.609) = 0.030, p = .970). There was a significant difference between conditions for total fat intake (*Welch's F* (2,80.868) = 4.326, p = .016). A post-hoc Games-Howell test indicated significantly lower total fat intake (grams) for the kiwifruit condition during the second half of the intervention (weeks 5 – 6) compared to the vitamin C condition (p = .016; **Supplemental Table 6**). There was also a trend for total carbohydrate intake (F (2,125) = 2.370, p = .098), driven by participants in the vitamin C condition consuming slightly more carbohydrates than participants in the placebo condition (p = .098).

As shown in the ANCOVA results in **Table 5** (main manuscript), most of the effects remained significant when adjusting for age, Asian ethnicity, year at university or polytechnic, lead-in sleep quality (weeks 1-2), fat intake during intervention (weeks 5-6), and carbohydrate intake during intervention (weeks 5-6). The condition x time effects comparing Week 2 and Week 4 were still significant for fatigue (F(2,119) = 3.514, p = .033, η_p^2 = 0.056) and well-being (F(2,119) = 5.012, p = .008, η_p^2 = 0.078) and were attenuated for POMS total mood disturbance (F(2,119) = 2.729, p = .069, η_p^2 = 0.044). There were no significant condition x time effects when comparing Study Week 2 (end of lead-in) with Study Week 6 (end of intervention) except for a trend for fatigue (fatigue F(2,119) = 2.842, p = .062, η_p^2 = 0.046; POMS total mood disturbance F(2, 119) = 1.830, p = .165, η_p^2 = 0.030; well-being (F(2,119) = 1.989, p = .141, η_p^2 = 0.032). Condition x time effects comparing Week 2, Week 4, and Week 6 were still significant for fatigue (F(2, 119) = 2.793, p = .027, η_p^2 = 0.045) and well-being (F(2,119) = 2.834, p = .025, η_p^2 = 0.045) but not for POMS total mood disturbance (F(2, 119) = 1.634, p = .166, η_p^2 = 0.027).

Supplemental Table 3. Participant characteristics for the below saturation vitamin C sample (*n* = 128) overall and by condition

	**Overall (*n* = 128)**	**Placebo (*n* = 45)**	**Vitamin C (*n* = 40)**	**Kiwifruit (*n* = 43)**
Age (years)	21.84 (3.56)	22.09 (3.15)	23.12 (4.66)	20.40 (2.01)
Gender:				
Male	46 (35.9%)	14 (31.1%)	16 (40.0%)	16 (37.2%)
Female	81 (63.3%)	31 (68.9%)	24 (60.0%)	26 (60.5%)
Gender diverse	1 (0.8%)	0 (0.0%)	0 (0.0%)	1 (2.3%)
Ethnicity:				
European	43 (33.6%)	12 (26.7%)	12 (30.0%)	19 (44.2%)
Asian	58 (45.3%)	25 (55.6%)	21 (52.2%)	12 (27.9%)
Indian	11 (8.6%)	3 (6.7%)	4 (10.0%)	4 (9.3%)
Māori & Pasifika	7 (5.5%)	2 (4.4%)	1 (2.5%)	4 (9.3%)
Other & multiple	9 (7.0%)	3 (6.7%)	2 (5.0%)	4 (9.3%)
Year of study at university	2.87 (1.54)	2.84 (1.51)	3.30 (1.80)	2.49 (1.20)
Socioeconomic status (1 to 7)[1]	4.64 (1.35)	4.78 (1.28)	4.51 (1.46)	4.60 (1.32)
Height (cm)	167.65 (10.13)	167.00 (10.86)	167.84 (9.45)	168.14 (10.17)
Weight (kg):				
Pre-intervention	67.06 (15.69)	67.19 (19.79)	66.64 (14.45)	67.33 (11.83)
Post-intervention[2]	68.30 (15.75)	67.479 (19.90)	67.31 (14.72)	67.12 (11.56)
BMI (kg/m2):				
Pre-intervention	23.77 (4.59)	23.99 (6.10)	23.51 (3.76)	23.77 (3.36)
Post-intervention[2]	23.85 (4.61)	24.09 (6.15)	23.74 (3.77)	23.70 (3.31)
No History of Smoking:	118 (92.2%)	42 (93.3%)	36 (90.0%)	40 (93.0%)
Vegetables servings/day	1.00 (0.71)	0.97 (0.84)	0.95 (0.67)	1.09 (0.79)
Fruit servings/day	0.36 (0.37)	0.35 (0.34)	0.35 (0.33)	0.39 (0.44)
Alcoholic standards /week	2.96 (4.84)	2.13 (3.89)	2.70 (5.09)	4.06 (5.37)
Sleep duration (hours)	7.16 (1.04)	7.10 (1.06)	7.36 (0.85)	7.06 (1.16)
Sleep quality (1 to 10)	6.56 (1.37)	6.20 (1.44)	6.83 (1.42)	6.69(1.18)
Physical activity (30 min/ day):				
% of days	44.5%	44.7%	47.0%	42.1%

Note. Values shown are means (SD) or *n* (%).[1] Based on participants who provided data (Total *n* = 127; Placebo *n* = 45; Vitamin C *n* = 40; Kiwifruit *n* = 42). [2] Based on participants who provided data (Total *n* = 127; Placebo *n* = 45; Vitamin C *n* = 39; Kiwifruit *n* = 43).

Supplemental Table 4. Below saturation vitamin C sample (n = 128) plasma vitamin C concentrations (µmol/L) by condition during the study period

	Placebo		Vitamin C		Kiwifruit	
	n	**Mean (SD)**	*n*	**Mean (SD)**	*n*	**Mean (SD)**
Week 0 (Lead-in)	45	29.09 (14.09)	40	28.06 (14.79)	43	34.91 (13.46)
Week 2 (Lead-in)	45	32.48 (15.29)	40	30.90 (15.82)	43	32.78 (14.00)
Week 4 (Intervention)	45	34.02 (17.95)	40	76.41 (18.45) **** [a]	43	78.33 (21.45) **** [a]
Week 6 (Intervention)	45	36.98 (20.67) *[a]	40	80.60 (17.92) **** [a]	43	76.25 (17.06) **** [a]
Week 8 (Washout)	44	35.06 (16.75)	40	52.53 (15.29) **** [abc]	41	47.78 (13.03) **** [abc]

Note. Results shown are means (SD). [a] Comparison with Week 2 (Lead-in); [b] Comparison with Week 4 (Intervention); [c] Comparison with Week 6 (Intervention). * $p < .05$, **** $p < .0001$.

Supplemental Table 5. Below saturation vitamin C sample (n = 128) raw mean scores (and standard deviations) of primary outcome measures and secondary covariates overall and by condition

	**Overall**		**Placebo**		**Vitamin C**		**Kiwifruit**	
	n	**Mean (SD)**	***n***	**Mean (SD)**	***n***	**Mean (SD)**	***n***	**Mean (SD)**
Primary Outcome Measures								
POMS TMD (range -20 to 100)								
Week 0 (Lead-in)	128	5.79 (14.48)	45	6.51 (12.65)	40	4.20 (15.49)	43	6.51 (15.49)
Week 2 (Lead-in)	128	5.27 (15.96)	45	3.33 (13.57)	40	5.05 (17.88)	43	7.49 (16.47)
Week 4 (Intervention)	128	4.23 (15.70)	45	5.78 (16.01)	40	2.42 (16.45)	43	4.28 (14.83) *[a]
Week 6 (Intervention)	128	3.98 (15.85)	45	4.53 (15.61)	40	1.68 (16.17)	43	5.53 (15.93)
Week 8 (Washout)	125	6.60 (17.57)	44	6.36 (17.45)	40	5.05 (19.33) *[c]	41	8.37 (16.12) *[bc]
Fatigue (range -24 to 96)								
Week 0 (Lead-in)	128	2.49 (14.28)	45	3.60 (13.41)	40	1.12 (15.78)	43	2.61 (13.93)
Week 2 (Lead-in)	128	1.55 (16.06)	45	0.82 (15.43)	40	1.51 (17.21)	43	2.34 (15.93)
Week 4 (Intervention)	128	0.13 (15.11)	45	2.38 (16.12)	40	-1.15 (16.59) †[a]	43	-1.03 (12.44) *[a]
Week 6 (Intervention)	128	0.47 (15.43)	45	1.80 (15.86)	40	-2.65 (15.78) *[a]	43	1.99 (14.55) *[b]
Week 8 (Washout)	125	1.91 (16.74)	44	2.79 (17.06)	40	0.54 (18.37) *[c]	41	2.30 (14.95) †[b]
Well-Being (range 14 to 70)								
Week 0 (Lead-in)	128	48.20 (8.49)	45	47.40 (8.38)	40	49.90 (8.94)	43	47.47 (8.14)
Week 2 (Lead-in)	128	48.48 (8.69)	45	49.24 (7.83)	40	48.78 (9.29)	43	47.42 (9.06)
Week 4 (Intervention)	128	48.43 (9.24)	45	46.69 (9.20) †[a]	40	49.14 (10.31)	43	49.60 (8.09) **[a]
Week 6 (Intervention)	128	49.73 (8.99)	45	48.91 (7.56) *[b]	40	51.18 (10.51) *[ab]	43	49.26 (8.87) †[a]
Week 8 (Washout)	125	49.04 (9.12)	44	47.91 (8.58)	40	49.85 (10.38)	41	49.47 (8.45) *[a]
Secondary Covariates								
Sleep Quantity (hours/night)								
Weeks 1-2 (Lead-in)	128	7.16 (1.04)	45	7.10 (1.06)	40	7.36 (0.85)	43	7.06 (1.16)
Weeks 3-4 (Intervention)	128	7.15 (0.98)	45	7.17 (1.02)	40	7.10 (0.90) †[d]	43	7.18 (1.02)
Weeks 5-6 (Intervention)	128	7.17 (1.01)	45	7.13 (0.98)	40	7.32 (1.08)	43	7.07 (0.97)
Weeks 7-8 (Washout)	127	7.03 (0.99)	45	6.90 (0.91)	40	6.96 (1.03) *[d]†[f]	42	7.24 (1.01)
Sleep Quality								
Weeks 1-2 (Lead-in)	128	6.56 (1.37)	45	6.20 (1.44)	40	6.83 (1.42)	43	6.69 (1.18)
Weeks 3-4 (Intervention)	128	6.61 (1.28)	45	6.45 (1.42) †[d]	40	6.82 (1.27)	43	6.58 (1.14)
Weeks 5-6 (Intervention)	128	6.72 (1.24)	45	6.56 (1.34) **[d]	40	6.91 (1.43)	43	6.71 (0.90)
Weeks 7-8 (Washout)	127	6.67 (1.30)	45	6.48 (1.37) **[d]	40	6.80 (1.35)	42	6.74 (1.18)
Physical Activity (% days active)								
Weeks 1-2 (Lead-in)	128	44.5%	45	44.7%	40	47.0%	43	42.1%
Weeks 3-4 (Intervention)	128	45.9%	45	45.4%	40	50.3%	43	42.2%
Weeks 5-6 (Intervention)	128	43.2%	45	44.6%	40	44.2% †[e]	43	40.8%
Weeks 7-8 (Washout)	127	44.2%	45	43.5%	40	49.1%	42	40.4%

Note. TMD = Total Mood Disturbance from the Profile of Mood States – Short Form Questionnaire. [a] Paired t-test comparison with Week 2 (Lead-in). [b] Paired t-test comparison to Week 4 (Intervention). [c] Paired t-test comparison with Week 6 (Intervention). [d] Paired t-test comparison with average smartphone survey responses during lead-in (Weeks 1-2). [e] Paired t-test comparison with average smartphone survey responses during intervention (Weeks 3-4). [f] Paired t-test comparison with average smartphone survey responses during intervention (Weeks 5-6). † $p < .10$, * $p < .05$, ** $p < .01$.

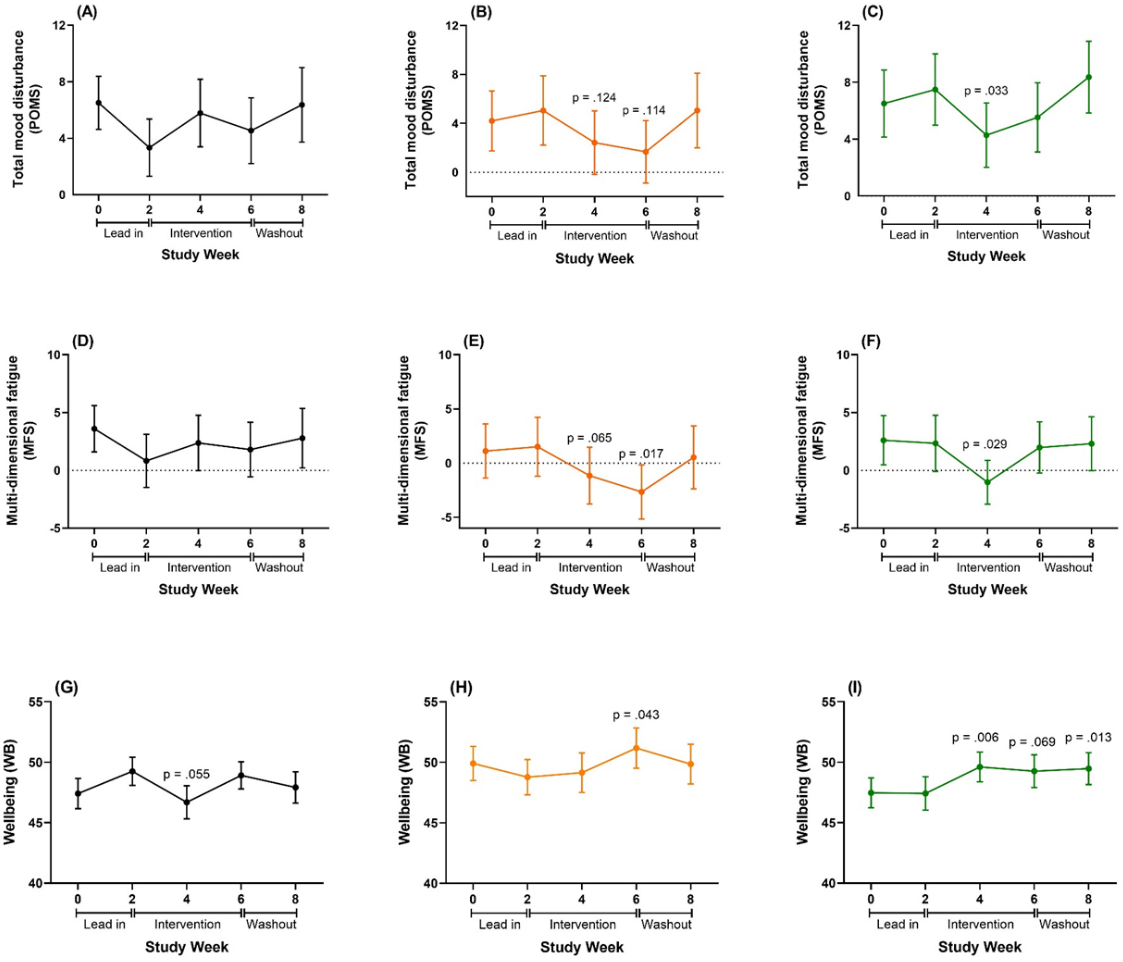

Supplemental Figure 1. Below saturation vitamin C sample (*n* = 128) changes in fortnightly measures of vitality [total mood disturbance (POMS), multidimensional fatigue score (MFS) and well-being (WB)] over the study period. Results are presented as raw unadjusted means ± SE for participants allocated to placebo tablet (black lines, A, D, G), vitamin C tablet (orange lines, B, E, H) and kiwifruit (green lines, C, F, I) condition. P values shown are derived from paired t-tests, comparing intervention points with the values at Lead-in Week 2 (end of lead-in). Where no p value is shown, results were not significantly different from lead-in values.

Supplemental Table 6. Below saturation vitamin C sample (*n* = 128) macronutrient diet record data for the placebo, vitamin C, and kiwifruit conditions

	**Placebo (*n* = 45)**		**Vitamin C (*n* = 40)**		**Kiwifruit (*n* = 43)**	
	n	**Mean (SD)**	***n***	**Mean (SD)**	***n***	**Mean (SD)**
Energy Intake (KJ)						
Week 1, Week 2 (Lead-in)	45	7880.04 (2734.68)	40	8350.63 (2751.51)	43	7945.32 (2421.31)
Week 5, Week 6 (Intervention)	45	7538.22 (3202.28)	40	8246.93 (2387.53)	43	7190.66 (2252.64) *[a]
Week 7, Week 8 (Washout)	44	6934.06 (2195.39) **[a]	39	7793.56 (2324.37)	40	7212.68 (1966.48) †[a]
Protein intake (grams)						
Week 1, Week 2 (Lead-in)	45	82.88 (33.53)	40	77.01 (28.72)	43	79.94 (37.42)
Week 5, Week 6 (Intervention)	45	78.39 (37.70)	40	76.81 (20.10)	43	76.96 (35.77)
Week 7, Week 8 (Washout)	44	71.45 (27.09) **[a]†[b]	39	73.71 (20.69)	40	73.52 (31.00)
Carbohydrate intake (grams)						
Week 1, Week 2 (Lead-in)	45	206.57 (83.86)	40	225.34 (79.80)	43	204.09 (65.07)
Week 5, Week 6 (Intervention)	45	192.08 (81.28)	40	226.43 (82.13)	43	199.16 (63.16)
Week 7, Week 8 (Washout)	44	183.20 (58.15) *[a]	39	212.01 (67.81)	40	181.99 (63.05) †[ab]
Fat intake (grams)						
Week 1, Week 2 (Lead-in)	45	78.86 (31.72)	40	86.11 (36.10)	43	81.54 (32.17)
Week 5, Week 6 (Intervention)	45	78.82 (45.76)	40	82.68 (29.55)	43	65.27 (26.15) **[a]
Week 7, Week 8 (Washout)	44	69.13 (30.07) *[a]	39	78.13 (30.81)	40	75.02 (24.44) *[b]

Note. Results shown are means (SD). KJ = kilojoules. [a] Comparison with Lead-in; [b] Comparison with Intervention.
† $p < .10$, * $p < .05$, ** $p < .01$.

Per Protocol Analysis Results (*n*= 92)

Subgroup per protocol (PP) analyses were conducted on 92 people who maintained a vitamin C level <40 μmol/L from screening until the beginning of the study (Week 0). There were few differences in demographic characteristics, baseline health characteristics, or baseline vitality measures between those 92 participants included in the PP analyses compared to the 75 participants excluded from these analyses (using Chi-Square statistics or t-tests, all not significant), except that the 92 included participants had higher baseline sleep quality (t(164) = 2.151, p = .033) and were more likely to be Asian (trend only) (χ^2 (1, 167) = 3.736, p = .053) than excluded participants.

Participant characteristics for this subgroup are shown in **Supplemental Table 7**. Of the 92 participants, 36 were in the placebo condition, 29 were in the vitamin C condition, and 27 were in the kiwifruit condition. The three conditions were mostly equivalent in demographic characteristics, baseline health characteristics, baseline vitality measures, and baseline vitamin C levels (all ANOVAs or Chi-Squares not significant). However, participants in the kiwifruit condition had younger participants (F(2,89) = 5.757, p = .004) who had completed fewer years of study (F(2,89) = 3.215, p = .045) than the other conditions. Plasma vitamin C concentrations during the study for PP participants are shown in **Supplemental Table 8**. Participants in the vitamin C and kiwifruit conditions achieved saturation in vitamin C after two weeks of supplementation (Week 4, Intervention).

The changes in the vitality outcomes by condition for the per protocol analyses (n = 92) are shown in **Supplemental Table 9** and **Supplemental Figure 2**. For the POMS total mood disturbance, participants in the vitamin C condition (**Supplemental Figure 2B**) showed significantly reduced total mood disturbance after two weeks of intervention (Study Week 4), but not at the end of the intervention (Study Week 6) or Washout (Study Week 8). Participants in the kiwifruit condition showed no significant reduction in their POMS total mood disturbance (**Supplemental Figure 2C**) and those in the placebo condition had a slight increase in total mood disturbance at washout in comparison to the end of lead-in (**Supplemental Figure 2A**). Participants in the vitamin C condition showed a significant reduction in fatigue after two weeks and a trend decrease in fatigue after four weeks of supplementation (**Supplemental Figure 2E**). For the kiwifruit condition, there was a non-significant reduction in fatigue after two weeks that returned to prior levels at four weeks (**Supplemental Figure 2F).** In the placebo condition, there was a trend increase in fatigue at washout when compared to the end of lead-in (**Supplemental Figure 2D)**. For well-being, there was a significant increase in well-being after two weeks of kiwifruit supplementation, which did not decrease at washout (**Supplemental Figure 2I**). Participants in the vitamin C group also showed a trend increase in well-being at the end of the intervention (**Supplemental Figure 2H**), which was eliminated at washout. There was no change in well-being throughout the study in the placebo group (**Supplemental Figure 2G**).

Analysis of variance (**Table 5**, main manuscript) showed significant condition x time effects when comparing Study Week 2 (end of lead-in) with Study Week 4 (second week of intervention) for POMS total mood disturbance (F(2, 89) = 3.841, p = .025, η_p^2 = 0.079), fatigue (F(2, 89) = 3.621, p = .031, η_p^2 = 0.075), and well-being (F(2, 189) = 3.611, p = .031, η_p^2 = 0.075). When comparing Study Week 2 (end of lead-in) with Study Week 6 (end of intervention), there was a significant effect for fatigue (F(2,89) = 3.113, p = .049, η_p^2 = 0.065), but not POMS total mood disturbance (F(2,89) = 2.099, p = .129, η_p^2 = 0.045)

or well-being (F(2,89) = 1.700, *p* = .189, η_p^2 = 0.037). When comparing Study Week 2, Week 4, and Week 6, the condition x time effects were significant for fatigue (F(2,89) = 3.021, *p* = .019, η_p^2 = 0.064) and showed trends for POMS total mood disturbance (F(2,89) = 2.262, *p* = .064, η_p^2 = 0.048) and well-being (F(2,89) = 2.088, *p* = .084, η_p^2 = 0.045).

The intervention effects in the PP analyses were not due to differences between conditions in sleep quantity (intervention weeks 3-4, F(2,89) = 0.061, *p* = .941; intervention weeks 5-6, F(2,89) = 0.590, *p* = .557), sleep quality (intervention weeks 3-4, F(2,89) = 1.293, *p* = .279; intervention weeks 5-6, F(2,89) = 1.203, *p* = .305), physical activity intervention weeks 5-6, F(2,89) = 0.315, *p* = .731), BMI (*Welch's F*(2,58.479) = 0.484, *p* = .619), or dietary factors such as total energy intake (KJ) (F(2,89) = 1.537, *p* = .221), total protein intake (*Welch's F*(2,55.251) = 0.292, *p* = .748), or total fat intake (*F*(2,89) = 1.115, *p* = .332). There was a significant difference between conditions for total carbohydrate intake (*F*(2,89) = 3.246, *p* = .044). A post-hoc Tukey test indicated significantly lower total carbohydrate intake (grams) for the placebo condition during the second half of the intervention (weeks 5 – 6) compared to the vitamin C condition (*p* = .034; **Supplemental Table 10**). There was also a trend difference in physical activity levels (intervention weeks 3-4, F(2,89) = 2.419, *p* = .095) driving by a participants in the vitamin C condition engaging in slightly more days with physical activity compared to the kiwifruit condition (*p* =.099; **Supplemental Table 9**).

When adjusting for age, year at university or polytechnic, physical activity during intervention (weeks 3-4), and carbohydrate intake during intervention (weeks 5-6), condition x time effects comparing Week 2 (Lead-in) and Week 4 (second week of intervention) remained significant for well-being (F(2,85) =3.590, *p* = .032, η_p^2 = 0.078) and were attenuated for POMS total mood disturbance (F(2,85) = 2.874, *p* = .062, η_p^2 = 0.063) and fatigue (F(2,85) = 2.992, *p* = .056, η_p^2 = 0.066). Results were attenuated for condition x time effects when comparing Study Week 2 (end of lead-in) with Study Week 6 (end of intervention) for fatigue (fatigue F(2,85) = 2.554, *p* = .084, η_p^2 = 0.057) and not significant for POMS total mood disturbance (F(2,85) = 1.270, *p* = .286, η_p^2 = 0.029) and well-being (F(2,85) = 1.463, *p* = .237, η_p^2 = 0.033). When comparing Week 2, Week 4, and Week 6, condition x time effects were significant for fatigue (F(2,85) = 2.638, *p* = .036, η_p^2 = 0.058) and attenuated for POMS total mood disturbance (F(2,85) = 1.635, *p* = .168, η_p^2 = 0.037) and well-being (F(2,85) = 1.981, *p* = .100, η_p^2 = 0.045). See **Table 5** in the main manuscript for full ANCOVA results.

Supplemental Table 7. Per protocol sample (*n* = 92) participant characteristics overall and by condition

	**PP Sample (*n* = 92)**	**Placebo (*n* = 36)**	**Vitamin C (*n* = 29)**	**Kiwifruit (*n* = 27)**
Age (years)	21.85 (3.73)	22.19 (3.21)	23.14 (5.01)	20.00 (1.49)
Gender:				
Male	30 (32.6%)	10 (27.8%)	11 (37.9%)	9 (33.3%)
Female	61 (66.3%)	26 (72.2%)	18 (62.1%)	17 (63.0%)
Gender diverse	1 (1.1%)	0 (0.0%)	0 (0.0%)	1 (3.7%)
Ethnicity:				
European	31 (33.7%)	8 (22.2%)	9 (31.0%)	14 (51.9%)
Asian	43 (46.7%)	19 (52.8%)	16 (55.2%)	8 (29.6%)
Indian	7 (7.6%)	4 (11.1%)	2 (6.9%)	1 (3.7%)
Māori & Pasifika	5 (5.4%)	2 (5.6%)	0 (0.0%)	3 (11.1%)
Other & multiple	6 (6.5%)	3 (8.3%)	2 (6.9%)	1 (3.7%)
Year of study at university	2.93 (1.53)	3.11 (1.51)	3.28 (1.85)	2.33 (0.92)
Socioeconomic status (1 to 7)[1]	4.73 (1.31)	4.84 (1.25)	4.7 (1.43)	4.6 (1.29)
Height (cm)	166.90 (9.69)	166.14 (10.68)	166.82 (9.17)	167.98 (9.1)
Weight (kg):				
Pre-intervention	66.66 (16.14)	66.15 (20.79)	67.63 (14.31)	66.28 (10.41)
Post-intervention[2]	66.86 (16.33)	66.38 (20.98)	68.27 (14.64)	66.03 (10.22)
BMI (kg/m2):				
Pre-intervention	23.85 (4.92)	23.84 (6.42)	24.18 (3.9)	23.52 (3.53)
Post-intervention[2]	23.93 (4.96)	23.92 (6.49)	24.42 (3.94)	23.43 (3.45)
No History of Smoking:	85 (92.4%)	34 (94.4%)	36 (89.7%)	25 (92.6%)
Vegetables servings/day	1.02 (0.73)	0.93 (0.64)	0.97 (0.71)	1.18 (0.86)
Fruit servings/day	0.38 (0.40)	0.38 (0.35)	0.35 (0.35)	0.4 (0.51)
Alcoholic standards /week	2.90 (4.83)	1.99 (3.77)	2.66 (5.07)	4.37 (5.6)
Sleep duration (hours)	7.19 (0.92)	7.10 (0.65)	7.47 (0.95)	7.02 (1.15)
Sleep quality (1 to 10)	6.74 (1.18)	6.51 (1.10)	6.94 (1.45)	6.82 (0.91)
Physical activity (30 min/ day):				
% of days	46.70%	44.30%	51.80%	44.40%

Notes. Values shown are means (standard deviations) or *n* (%).[1] Based on participants who provided data (Total *n* = 91; Placebo *n* = 36; Vitamin C *n* = 29; Kiwifruit *n* = 26). [2] Based on participants who provided data (Total *n* = 91; Placebo *n* = 36; Vitamin C *n* = 28; Kiwifruit *n* = 27).

Supplemental Table 8. Per protocol sample (*n* = 92) plasma vitamin C concentrations (μmol/L) by condition during the study period

	Placebo		Vitamin C		Kiwifruit	
	n	**Mean (SD)**	*n*	**Mean (SD)**	*n*	**Mean (SD)**
Week 0 (Lead-in)	36	23.64 (9.47)	29	20.77 (9.50)	27	26.60 (8.70)
Week 2 (Lead-in)	36	28.71 (14.13)	29	25.03 (13.39)	27	27.67 (13.26)
Week 4 (Intervention)	36	29.70 (16.65)	29	78.43 (20.57) ****[a]	27	78.27 (23.09) ****[a]
Week 6 (Intervention)	36	33.94 (21.23) *[a]†[b]	29	80.83 (20.07) ****[a]	27	77.19 (17.98) ****[a]
Week 8 (Washout)	35	32.23 (16.40)	29	50.53 (15.61) ****[abc]	25	46.85 (14.09) ****[abc]

Note. Results shown are means and standard deviations (SD). [a] Comparison with Week 2 (Lead-in); [b] Comparison with Week 4 (Intervention); [c] Comparison with Week 6 (Intervention). † $p < .10$, * $p < .05$, **** $p < .0001$.

Supplemental Table 9. Per protocol sample (*n* = 92) raw mean scores (and standard deviations) of primary outcome measures and secondary covariates overall and by condition

	Overall		Placebo		Vitamin C		Kiwifruit	
	n	Mean (SD)	*n*	Mean (SD)	*n*	Mean (SD)	*n*	Mean (SD)
Primary Outcome Measures								
POMS TMD (range -20 to 100)								
Week 0 (Lead-in)	92	4.85 (13.55)	36	5.42 (12.35)	29	2.21 (14.12)	27	6.93 (14.48)
Week 2 (Lead-in)	92	3.86 (15.34)	36	1.33 (11.42)	29	3.90 (18.19)	27	7.19 (16.48)
Week 4 (Intervention)	92	3.59 (15.85)	36	5.19 (16.17)	29	-0.24 (14.36) *[a]	27	5.56 (16.77)
Week 6 (Intervention)	92	3.46 (15.38)	36	3.58 (13.48)	29	0.48 (16.52)	27	6.48 (16.45)
Week 8 (Washout)	89	5.56 (17.23) *[c]	35	5.31 (16.26) *[a]	29	3.45 (19.65) †[b]	25	8.36 (15.79)
Fatigue (range -24 to 96)								
Week 0 (Lead-in)	92	1.44 (13.98)	36	2.38 (13.58)	29	-1.10 (14.73)	27	2.91 (13.82)
Week 2 (Lead-in)	92	0.26 (14.89)	36	-1.08 (11.83)	29	0.18 (17.5)	27	2.15 (15.88)
Week 4 (Intervention)	92	-1.15 (14.48)	36	0.72 (15.09)	29	-3.83 (14.86) *[a]	27	-0.78 (13.27)
Week 6 (Intervention)	92	-0.17 (15.27)	36	0.33 (13.65)	29	-4.17 (16.22) †[a]	27	3.46 (15.81) *[b]
Week 8 (Washout)	89	1.15 (16.52) *[b]	35	1.56 (16.41) †[a]	29	-0.68 (18.04) †[bc]	25	2.68 (15.26)
Well-Being (range 14 to 70)								
Week 0 (Lead-in)	92	48.83 (8.44)	36	48.08 (8.20)	29	51.38 (8.82)	27	47.07 (8.00)
Week 2 (Lead-in)	92	48.93 (8.06)	36	49.67 (7.58)	29	50.21 (9.01)	27	46.59 (7.38)
Week 4 (Intervention)	92	49.08 (8.84)	36	47.67 (9.16)	29	50.95 (9.94)	27	48.96 (6.90) *[a]
Week 6 (Intervention)	92	50.03 (8.66)	36	49.14 (6.93)	29	52.48 (10.66) †[a]	27	48.59 (8.10)
Week 8 (Washout)	89	49.33 (8.57)	35	48.31 (7.71)	29	51.00 (10.21)	25	48.81 (7.65) *[a]
Secondary Covariates								
Sleep Quantity (hours/night)								
Weeks 1-2 (Lead-in)	92	7.19 (0.92)	36	7.10 (0.65)	29	7.47 (0.95)	27	7.02 (1.15)
Weeks 3-4 (Intervention)	92	7.14 (0.92)	36	7.10 (0.91)	29	7.19 (1.00)	27	7.13 (0.88)
Weeks 5-6 (Intervention)	92	7.21 (0.97)	36	7.12 (0.92)	29	7.37 (1.19)	27	7.14 (0.74)
Weeks 7-8 (Washout)	91	7.04 (1.00)	36	6.94 (0.84)	29	6.96 (1.03) *[d]†[f]	26	7.28 (1.16)
Sleep Quality								
Weeks 1-2 (Lead-in)	92	6.74 (1.18)	36	6.51 (1.10)	29	6.94 (1.45)	27	6.82 (0.91)
Weeks 3-4 (Intervention)	92	6.74 (1.15)	36	6.61 (1.04)	29	7.02 (1.32)	27	6.60 (1.07)
Weeks 5-6 (Intervention)	92	6.84 (1.09)	36	6.73 (0.97)	29	7.10 (1.36)	27	6.72 (0.90)
Weeks 7-8 (Washout)	91	6.87 (1.13)	36	6.81 (0.91) *[d]	29	6.91 (1.35)	26	6.92 (1.16)
Physical Activity (% days active)								
Weeks 1-2 (Lead-in)	92	46.7%	36	44.3%	29	51.8%	27	44.4%
Weeks 3-4 (Intervention)	92	46.7%	36	43.9%	29	56.7%	27	39.6%
Weeks 5-6 (Intervention)	92	43.0%	36	43.5%	29	46.2% *[e]	27	39.1%
Weeks 7-8 (Washout)	91	47.2%	36	42.9%	29	54.1% †[f]	26	45.4%

Note. TMD = Total Mood Disturbance from the Profile of Mood States – Short Form Questionnaire. [a] Paired t-test comparison with Week 2 (Lead-in). [b] Paired t-test comparison with Week 4 (Intervention). [c] Paired t-test comparison with Week 6 (Intervention)). [d] Paired t-test comparison with average smartphone survey responses during Weeks 1-2 (Lead-in). [e] Paired t-test comparison with average smartphone survey responses during Weeks 3-4 (Intervention). [f] Paired t-test comparison with average smartphone survey responses during Weeks 5-6 (Intervention). † $p < .10$, * $p < .05$, ** $p < .01$.

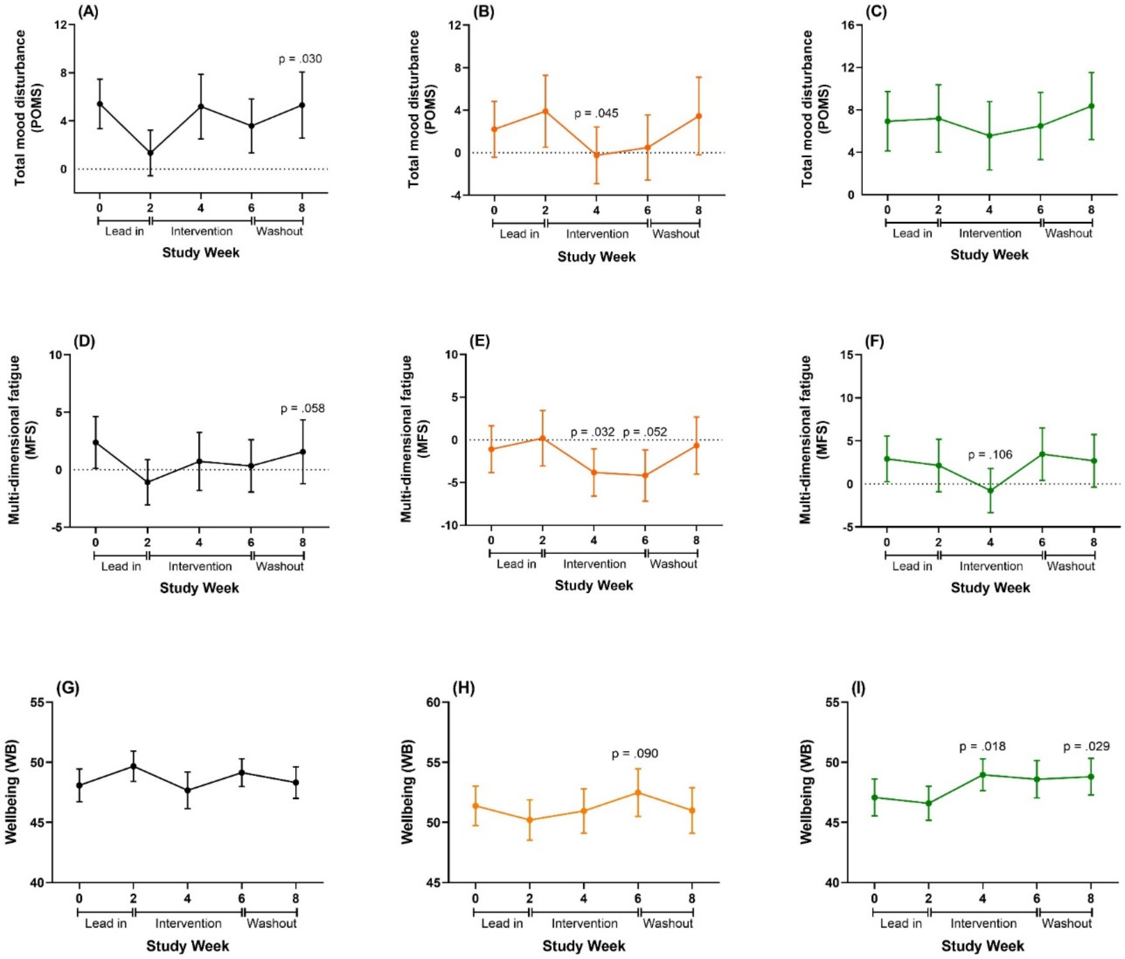

Supplemental Figure 2. Per protocol sample (*n* = 92) changes in fortnightly measures of vitality [total mood disturbance (POMS), multidimensional fatigue score (MFS) and well-being (WB)] over the study period. Results are presented as raw unadjusted means ± SE for individuals allocated to the placebo tablet (black lines, A, D, G), vitamin C tablet (orange lines, B, E, H) and kiwifruit (green lines, C, F, I) condition. P values shown are derived from paired t-tests, comparing intervention points with the values at Lead-in Week 2 (end of lead-in). Where no p value is shown, results were not significantly different from lead-in values.

Supplemental Table 10. Per protocol sample (*n* = 92) macronutrient diet record data for the placebo, vitamin C, and kiwifruit conditions

	Placebo (n = 36)		Vitamin C (n = 29)		Kiwifruit (n = 27)	
	n	Mean (SD)	*n*	Mean (SD)	*n*	Mean (SD)
Energy Intake (KJ)						
Week 1, Week 2 (Lead-in)	36	7580.90 (2569.73)	29	8403.28 (3000.45)	27	8175.59 (2002.77)
Weeks 5-6 (Intervention)	36	7083.73 (2980.05)	29	8205.30 (2510.72)	27	7346.48 (2225.20) †[a]
Weeks 7-8 (Washout)	36	6666.06 (2071.07) *[a]	28	7971.27 (2526.62)	24	7602.07 (2134.94)
Protein intake (grams)						
Weeks 0-2 (Lead-in)	36	78.63 (30.94)	29	75.69 (30.11)	27	79.88 (30.26)
Weeks 4-6 (Intervention)	36	73.11 (32.69)	29	75.88 (18.26)	27	79.21 (29.97)
Weeks 6-8 (Washout)	36	67.24 (21.90) *[a]	28	74.26 (23.09)	24	71.98 (24.06)
Carbohydrate intake (grams)						
Weeks 0-2 (Lead-in)	36	198.38 (68.28)	29	224.15 (86.18)	27	216.20 (59.34)
Weeks 4-6 (Intervention)	36	182.82 (76.50)	29	231.34 (90.60)	27	207.63 (57.57)
Weeks 6-8 (Washout)	36	173.87 (54.79) *[a]	28	216.87 (72.93)	24	197.79 (69.10)
Fat intake (grams)						
Weeks 0-2 (Lead-in)	36	76.37 (31.01)	29	88.58 (38.81)	27	81.04 (22.78)
Weeks 4-6 (Intervention)	36	73.04 (41.68)	29	80.09 (29.03)	27	66.46 (27.28) *[a]
Weeks 6-8 (Washout)	36	67.85 (29.38) †[a]	28	80.06 (33.56)	24	79.18 (26.77) †[b]

Note. Results shown are means (SD). KJ = kilojoules. [a] Paired t-test comparison with Lead-in; [b] Paired t-test comparison with Intervention. † $p < .1$, * $p < .05$, ** $p < .01$.

Sensitivity Analysis using Pattern Mixture Models

Pattern mixture models were used to test the robustness of assumptions for the primary analysis, assessing the plausible impact of data missing not at random (MNAR). Patterns in a pattern mixture model represent different groups of missing data. It is thought participants who drop out of a study may have a different distribution from those who remain in the study and have observed data. Different patterns of missing data can be classified by the time point in which participants dropped out, by participant reasons for dropping out, or auxiliary variables collected at baseline. We do acknowledge that this type of sensitivity analysis cannot assess all possibilities for missing data, as it is limited by possible under-identification of subgroups that can explain the missing data, as data required to determine these subgroups may be missing. However, by definition, these possible subgroups are untestable. A strength of the current study is that at baseline we collected data on several auxiliary variables, such as age, gender, ethnicity, smoking status, year of study, typical weekly alcohol consumption, weight, fruit and vegetable consumption, and socioeconomic status, which were used to determine differences between participants with observed and missing data on primary outcomes.

For the primary outcomes, missing data followed a monotone pattern. If data was missing at a time point (k), then data would also be missing at the following time point(s) (k+1). As such, a pattern mixture approach accounting for the probability of drop out over time was taken (**Supplemental Table 11**). Recommendations and equations were taken from the panel of handling missing data in clinical trials for monotone missing data; National Research Council, 2010.

Supplemental Table 11. Discontinued participants (cumulative percentage (*n*))

Visit	Study Phase	Placebo (*n* = 54)	Vitamin C (*n* = 56)	Kiwifruit (*n* = 57)
Visit 1	Week 0 (Lead-in)	0.0% (0)	0.0% (0)	0.0% (0)
Visit 2	Week 2 (Lead-in)	3.7% (2)	1.8% (1)	0.0% (0)
Visit 3	Week 4 (Intervention)	3.7% (2)	5.4% (3)	1.8% (1)
Visit 4	Week 6 (Intervention)	3.7% (2)	7.1% (4)	3.5% (2)
Visit 5	Week 8 (Washout)	5.6% (3)	8.9% (5)	7.0% (4)

$$E(Y_0 \mid L \geq 1) = \mu_0, \quad (1)$$

$$E(Y_1 \mid Y_0, L \geq 2) = \mu_1 + \beta_1 Y_0, \quad (2)$$

$$E(Y_2 \mid Y_0, Y_1, L \geq 3) = \mu_2 + \beta_2 (Y_0, Y_1)^T, \quad (3)$$

$$E(Y_3 \mid Y_0, Y_1, Y_2, L \geq 4) = \mu_3 + \beta_3 (Y_0, Y_1, Y_2)^T, \quad (4)$$

$$E(Y_4 \mid Y_0, Y_1, Y_2, Y_3, L = 5) = \mu_4 + \beta_4 (Y_0, Y_1, Y_2, Y_3)^T. \quad (5)$$

The above equations show the regression model estimates for observed data on the continuous primary outcomes for each of the time points ((1) Week 0 (Lead-in), (2) Week 2 (Lead-in), (3) Week 4 (Intervention), (4) Week 6 (Intervention), (5) Week 8 (Washout) based on the relationship between Y_k and Y_{k-1} for all available cases, where Y_k is the outcome (total mood disturbance, fatigue, or well-being) at time point k (e.g. when assessing total mood disturbance (TMD), Y_4, would correspond to TMD at Week 8 (washout)). The last week in which as participant provided data is denoted as L. The outcome mean at each week from observed data is denoted as μ_k, where $\mu = E(Y_k)$ and β is the slope coefficient from the given regression model. Similar regression equations are used to estimate missing data under different patterns of missingness.

$$E(Y_1 \mid Y_0, L = 1) = \mu_1^* + \beta_1^* Y_0, \quad (6)$$

$$E(Y_2 \mid Y_0, Y_1, L \geq 3) = \mu_2^* + \beta_2^* (Y_0, Y_1)^T, \quad (7)$$

$$E(Y_3 \mid Y_0, Y_1, Y_2, L \geq 4) = \mu_3^* + \beta_3^* (Y_0, Y_1, Y_2)^T, \quad (8)$$

$$E(Y_4 \mid Y_0, Y_1, Y_2, Y_3, L = 5) = \mu_4^* + \beta_4^* (Y_0, Y_1, Y_2, Y_3)^T. \quad (9)$$

Where $\mu_k^* = \mu_k + \Delta_{\mu k}$ and $\beta_k^* = \beta_k + \Delta_{\beta k}$. The $\Delta_{\mu k}$being the difference between the mean of Y_k for individuals who drop out at k-1 and individuals remained in the study at k-1. The same principle can be used to determine $\Delta_{\beta k}$, however, to simplify the number of sensitivity parameters, $\Delta_{\beta k}$= 0, assuming that $\Delta_{\mu k}$ is similar between patterns.

There was no significant difference between participants who remained in the study and participants who dropped out for age, gender, smoking status, typical weekly alcohol consumption, weight, fruit and vegetable consumption, or socioeconomic status. However, there were differences in ethnicity, in which more non-Asian participants dropped out than Asian participants and participants who had completed fewer years of study were more likely to drop out. **Supplemental Table 12** shows the ANOVA results for sensitivity analysis under different patterns of missingness. Pattern one assumes that data is missing at random (MAR, Δ=0), considering drop out over time. Pattern two assumes that

there are differences between conditions for participants who drop out over time. Pattern three grouped participants who dropped out based on their vitamin C levels into four groups; unhealthy levels of vitamin C (<23µmol/L), adequate vitamin C, (23-40µmol/L), healthy vitamin C (41-60µmol/L), and saturated (>60µmol/L), conditional on condition and time of drop out. Pattern four grouped participants based on their reason for withdrawal or dropout over time. Groupings based on reasons for drop out included medical or health reasons, no contact / no show, and disliked assigned condition and unwilling to adhere. Pattern five addresses the ethnicity discrepancies between conditions over time. Pattern six addresses the year of study differences between participants who dropped out and participants who completed the study.

Sensitivity analysis (**Supplemental Table 12**) overall indicated that missing data was mostly negligible, and analyses were robust as the patterns of results remained the same or were marginally strengthened with the increased power. Sensitivity analysis for patterns one, two, three, four, and six gave comparable results to the primary analysis for all vitality outcomes, although they were slightly strengthened. Pattern five results were strengthened for total mood disturbance, fatigue, and well-being, which may suggest that there is an ethnicity-linked difference between conditions. Ethnicity-linked differences between vitamin C levels and vitality outcomes have also been noted in the main analysis and may require further investigation. However, our study was under-powered to fully explore such effects.

Reference

National Research Council. *The prevention and treatment of missing data in clinical trials*. Panel on Handling Missing Data in Clinical Trials. Committee on National Statistics, Division of Behavioral and Social Sciences and Education. Washington, DC: The National Academies Press; 2010

Supplemental Table 12. Sensitivity analysis using pattern mixture models

		**Total Mood Disturbance (*n*= 167)**			**Fatigue (*n* = 167)**			**Well-being (*n* = 167)**		
Pattern	Time	F	p	η_p^2	F	p	η_p^2	F	p	η_p^2
Pattern One	Week 2, Week 4	3.361	.037	0.039	2.897	.058	0.034	4.586	.012	0.053
	Week 2, Week 6	1.805	.168	0.022	0.868	.422	0.010	2.467	.088	0.029
	Week 2, Week 4, Week 6	1.789	.131	0.021	1.400	.234	0.017	2.496	.043	0.030
Pattern Two	Week 2, Week 4	3.900	.022	0.045	2.945	.055	0.035	4.787	.010	0.055
	Week 2, Week 6	1.334	.266	0.016	0.456	.634	0.006	2.302	.103	0.027
	Week 2, Week 4, Week 6	1.805	.128	0.022	1.329	.259	0.016	2.724	.029	0.032
Pattern Three	Week 2, Week 4	3.890	.022	0.045	3.269	.041	0.038	4.557	.012	0.053
	Week 2, Week 6	1.420	.245	0.017	0.491	.613	0.006	1.996	.139	0.024
	Week 2, Week 4, Week 6	1.887	.115	0.023	1.427	.225	0.017	2.624	.035	0.031
Pattern Four	Week 2, Week 4	3.314	.039	0.039	2.750	.067	0.032	4.777	.010	0.056
	Week 2, Week 6	1.295	.277	0.016	0.450	.638	0.005	2.441	.090	0.029
	Week 2, Week 4, Week 6	1.639	.164	0.020	1.226	.300	0.015	2.727	.029	0.033
Pattern Five	Week 2, Week 4	4.316	.015	0.050	3.406	.036	0.040	5.094	.007	0.058
	Week 2, Week 6	2.927	.056	0.034	0.711	.493	0.009	2.860	.060	0.034
	Week 2, Week 4, Week 6	2.449	.046	0.029	1.505	.200	0.018	2.934	.021	0.035
Pattern Six	Week 2, Week 4	4.298	.015	0.050	2.819	.063	0.033	3.938	.021	0.046
	Week 2, Week 6	1.771	.173	0.021	0.789	.456	0.010	1.826	.164	0.022
	Week 2, Week 4, Week 6	2.075	.084	0.025	1.339	.255	0.016	2.178	.071	0.026

Note. Pattern one: Missing at random (MAR, Δ=0); Pattern two: Different distributions between conditions; Pattern three: Distributions based on vitamin C levels; Pattern four: Distributions based on reason for withdrawal or dropout; Pattern five: Distributions based on ethnicity discrepancies between conditions; Pattern six: Distributions based on year of study.
